# Exploring non-invasive precision treatment in non-small cell lung cancer patients through deep learning radiomics across imaging features and molecular phenotypes

**DOI:** 10.1186/s40364-024-00561-5

**Published:** 2024-01-25

**Authors:** Xingping Zhang, Guijuan Zhang, Xingting Qiu, Jiao Yin, Wenjun Tan, Xiaoxia Yin, Hong Yang, Hua Wang, Yanchun Zhang

**Affiliations:** 1 School of Medical Information Engineering, Gannan Medical University, 341000 Ganzhou, China; 2 Cyberspace Institute of Advanced Technology, Guangzhou University, 510006 Guangzhou, China; 3 Department of Respiratory and Critical Care, First Affiliated Hospital of Gannan Medical University, 341000 Ganzhou, China; 4 School of Computer Science and Technology, Zhejiang Normal University, 321000 Jinhua, China; 5 Institute for Sustainable Industries and Liveable Cities, Victoria University, 3011 Melbourne, Australia; 6 Department of New Networks, Peng Cheng Laboratory, 518000 Shenzhen, China; 7 Department of Radiology, First Affiliated Hospital of Gannan Medical University, 341000 Ganzhou, China; 8 Key Laboratory of Intelligent Computing in Medical Image, Ministry of Education, Northeastern University, 110189 Shenyang, China

**Keywords:** Deep learning, Radiomics, Actionable mutations, Immune status, Targeted therapy and immunotherapy, NSCLC

## Abstract

**Background:**

Accurate prediction of tumor molecular alterations is vital for optimizing cancer treatment. Traditional tissue-based approaches encounter limitations due to invasiveness, heterogeneity, and molecular dynamic changes. We aim to develop and validate a deep learning radiomics framework to obtain imaging features that reflect various molecular changes, aiding first-line treatment decisions for cancer patients.

**Methods:**

We conducted a retrospective study involving 508 NSCLC patients from three institutions, incorporating CT images and clinicopathologic data. Two radiomic scores and a deep network feature were constructed on three data sources in the 3D tumor region. Using these features, we developed and validated the ‘Deep-RadScore,’ a deep learning radiomics model to predict prognostic factors, gene mutations, and immune molecule expression levels.

**Findings:**

The Deep-RadScore exhibits strong discrimination for tumor molecular features. In the independent test cohort, it achieved impressive AUCs: 0.889 for lymphovascular invasion, 0.903 for pleural invasion, 0.894 for T staging; 0.884 for EGFR and ALK, 0.896 for KRAS and PIK3CA, 0.889 for TP53, 0.895 for ROS1; and 0.893 for PD-1/PD-L1. Fusing features yielded optimal predictive power, surpassing any single imaging feature. Correlation and interpretability analyses confirmed the effectiveness of customized deep network features in capturing additional imaging phenotypes beyond known radiomic features.

**Interpretation:**

This proof-of-concept framework demonstrates that new biomarkers across imaging features and molecular phenotypes can be provided by fusing radiomic features and deep network features from multiple data sources. This holds the potential to offer valuable insights for radiological phenotyping in characterizing diverse tumor molecular alterations, thereby advancing the pursuit of non-invasive personalized treatment for NSCLC patients.

**Supplementary Information:**

The online version contains supplementary material available at 10.1186/s40364-024-00561-5.

## Introduction

Lung cancer stands as one of the most prevalent and deadly malignancies globally. The 5-year survival rate for non-small cell lung cancer (NSCLC), which accounts for about 85% of cases, remains below 20% [1]. Although several strategies have fundamentally changed the treatment paradigm for cancer, overall survival rates have seen limited improvements. Within the NSCLC patient population, there is considerable variability in treatment responses and prognoses, even among individuals with the same type of tumor. This variability is primarily attributed to intra-tumor heterogeneity and patient-specific factors. Personalized medicine plays a crucial role in improving treatment outcomes and patient survival by tailoring therapies to the unique characteristics of each patient’s cancer.

Targeted therapy and immunotherapy are primary treatments for advanced NSCLC [2, 3]. The NCCN guidelines [4] recommend determining the status of several driver oncogenes before initiating treatment to assess the suitability of targeted agents that can improve survival, such as EGFR, KRAS, ALK, and ROS1. For patients without targeted therapeutic options, immune checkpoint inhibitors targeting PD-1/PD-L1 are increasingly used in therapy and have demonstrated positive results in significantly extending patient survival [5, 6]. Despite developing state-of-the-art targeted therapy and immunotherapy, only a small percentage of patients respond [7]. Additionally, lymphovascular invasion (LVI) is a substantial risk factor for patients with early-stage NSCLC. Preoperative neoadjuvant chemotherapy has yielded better long-term survival benefits among LVI-positive patients [8]. Pleural invasion (PI) may adversely affect the staging and treatment of NSCLC patients and may serve as an independent predictor of disease-free and overall survival [9]. Therefore, accurately detecting known risk factors, predictive biomarkers, and PD-L1/PD-L1 expression levels is crucial for developing personalized treatment strategies.

Traditionally, the detection of the aforementioned molecular alterations has relied on tissue samples obtained through biopsy or surgery [10–12]. Unfortunately, these known biomarkers can only partially capture the variability of results due to tumor biology and microenvironmental interactions. They are also limited by insufficient sample quantity or quality, intra-tumor heterogeneity, and patient discomfort. In contrast, radiological imaging has become routinely used in clinical practice for cancer screening, staging, assessing therapy response, and monitoring disease recurrence. It can noninvasively provide comprehensive information about the tumor and its surrounding parenchyma. Furthermore, medical images offer insights into the unique phenotypes resulting from the underlying biological processes of a tumor, which can be extracted for high-throughput quantitative characterization through radiomics [13, 14].

Conventional radiomic analysis assesses features extracted from regions of interest (ROIs) using statistical methodologies and machine learning techniques. This method is widely employed to predict tumor heterogeneity and various molecular features across diverse cancer types, including high-risk prognostic factors and predictive and immune-related biomarkers [15–17]. These radiomics studies have focused on analyzing comprehensive information about tumors’ radiological phenotype and microenvironmental heterogeneity using numerous quantitative features in the tumor region. Previous studies have also demonstrated that, in addition to the tumor region, the peritumoral region can provide additional critical information. Despite its utility, radiomics presents inherent limitations, notably the intricate ROI segmentation process and lack of category representation for hard-coded features. Recent developments underscore the emergence of an innovative paradigm by combining deep learning with radiomics [12, 18, 19]. Deep neural networks directly extract features, providing intricate, category-specific structural insights. Recent strides in this field have demonstrated the efficacy of deep network features in predicting tumor characteristics, therapeutic response, and overall prognosis in lung, breast, glioma, and rectal cancers [20, 22]. However, the construction of such imaging features, while advantageous for its efficiency and reduced labor intensity, grapples with challenges associated with small sample sizes, particularly impactful when addressing rare diseases. The primary challenge shared by both radiomics and deep learning approaches lies in the quantitative fusion of medical data from various modalities. This multifaceted effort aims to yield complementary phenotypic insights from disparate perspectives, culminating in heightened precision and reliability in outcome predictions.

Specific radiomics studies typically revolve around one imaging signature and one molecular signature. While selecting the preferred imaging modality for each disease being analyzed is necessary, integrating image biomarkers derived from specific molecular feature cohorts into the clinical management of cancer patients has been challenging, thereby limiting their clinical utility. In contrast to radiomics approaches that focus on one imaging feature and one molecular feature, strategies that work across imaging features and molecular features may lead to the identification of novel imaging phenotypes that are more compatible with clinical treatment, offering broad implications for the therapeutic management of cancer patients.

In this article, we aim to extract novel radiological features from routine CT scans carefully designed to characterize imaging phenotypes of different molecular alterations and complement known molecular biomarkers. Based on a multi-institutional cohort across radiological risk factors, gene mutation status, and PD-1/PD-L1 expression levels, we develop and validate a deep learning radiomics architecture that incorporates multiple data sources to comprehensively characterize different molecular alterations in individual patients and demonstrate its potential clinical value in guiding personalized treatment.

## Materials and methods

### Participant cohorts

We collected patient data from three institutions to discover and validate the imaging phenotype of molecular features. Cohort I consisted of 370 patients treated between October 2017 and September 2021 at the First Affiliated Hospital of Gannan Medical University. Clinical information, radiology reports, genetic testing reports, and immunohistochemistry testing reports were obtained from electronic case databases for Cohort I. CT scan data were retrieved from picture archiving and communication systems. The institutional ethical review board approved the retrospective study and waived the requirement for informed consent from patients.

Data from Cohort II, comprising 138 patients from the Palo Alto Veterans Affairs Healthcare System and Stanford University School of Medicine, is publicly available. This cohort’s data collection followed similar inclusion and exclusion criteria. However, the cohort did not include data on PD-1/PD-L1 expression, but RNA sequencing data were collected from patients. Previous studies by Sun et al. [23] and Tumeh et al. [24] have highlighted that CD8 cell infiltration status represents a priority for PD-1 expression. To ensure consistency in definitions, we defined PD-1/PD-L1 expression as a Tumor Proportion Score (TPS) $$ \ge $$ 1% or high CD8 cell infiltration. Conversely, PD-1/PD-L1 non-expression was defined as a TPS $$ <$$ 1% or low CD8 cell infiltration. For further details regarding inclusion criteria, exclusion criteria, and the recruitment process, please refer to Appendix E1 and Fig. S1.

Table 1 presents a comprehensive summary of the clinicopathological characteristics exhibited by all patients. Cohort I and II were combined into a new dataset to balance demographic and clinicopathologic factors distribution. The training and independent test cohorts were allocated in a 7:3 ratio for model development and testing.

### CT image preprocessing

CT images of patients within Cohort I were acquired using two distinct imaging scanners. The process of tumor ROI segmentation for each case was undertaken manually within the 3D Slicer v4.11 software platform. A clinician with five years of relevant experience performed the initial segmentation. Following this, a radiologist possessing ten years of specialized expertise conducted individual reviews and subsequent corrections to the segmented ROIs. When disparities or ambiguities arose in the segmentation results, resolution was achieved through a consultative assessment to reach a consensus. In contrast, the CT images and corresponding ROIs for Cohort II were procured from publicly accessible databases, thus facilitating additional evaluation.

In addition to the tumor regions, we expanded our dataset with two additional data sources. The first source is based on delineated tumor ROIs, automatically generated to outline the peritumoral region through an internal algorithm. This algorithm constructs a peripheral ring with a consistent thickness of 3 mm along the tumor boundary and designates it as peritumoral ROIs. The second data source consists of deep ROIs, wherein we carefully selected representative slices containing the largest ROI. These selections were used to create 160 × 160 pixel square image plaques centered on the centroid of the tumor ROIs. The plaque size was determined after considering the size of all tumors to ensure comprehensive coverage of the entire tumor area.

**Table 1:** Clinical characteristics of patients with NSCLC

Characteristics	Cohort I	Cohort II	***p*** value	Mutations	Training	Test	***p*** value
Number	370 (72.8%)	138 (27.2%)	-	EGFR			0.203
Age (years)			∗	Mutant	130 (38.5%)	56 (38.6%)	
mean ± SD	62.6 $\pm$ 10.7	69.2 $\pm$ 8.9		Wildtype	207 (61.5%)	89 (61.4%)	
Gender			*	KRAS			0.081
Male	229 (61.9%)	102 (73.9%)		Mutant	34 (10.3%)	23 (16.3%)	
Female	141 (38.1%)	36 (26.1%)		Wildtype	295 (89.7%)	118 (83.7%)	
Smoking status			*	ALK			0.124
Smoker	178 (48.1%)	116 (84.1%)		Mutant	14 (4.2%)	13 (9.0%)	
Nonsmoker	192 (51.9%)	22 (15.9%)		Wildtype	317 (95.8%)	130 (91.0%)	
Tumor location			0.379	TP53			0.904
RUL	110 (29.7%)	50 (36.2%)		Mutant	62 (22.9%)	58 (58.0%)	
RML	34 (9.2%)	12 (8.7%)		Wildtype	208 (77.1%)	42 (42.0%)	
RLL	68 (18.4%)	21 (15.2%)		PIK3CA			0.083
LUL	110 (29.7%)	35 (25.4%)		Mutant	28 (10.8%)	19 (17.1%)	
LLL	48 (13.0%)	20 (14.5%)		Wildtype	231 (89.2%)	92 (82.9%)	
**Risk factors**	**Training**	**Test**	***p*** **value**	ROS1			0.101
LVI			0.792	Mutant	16 (6.6%)	10 (8.7%)	
Present	114 (32.1%)	49 (32.0%)		Wildtype	240 (93.4%)	104 (91.3%)	
Absent	241 (67.9%)	104 (68.0%)		**Immune**	**Training**	**Test**	***p*** **value**
PI			0.139	PD-1/PD-L1			0.059
Yes	224 (63.1%)	96 (62.7%)		Positive	63 (50.4%)	22 (40.0%)	
No	131 (36.9%)	57 (37.3%)		Negative	62 (49.6%)	33 (60.0%)	
T staging			0.612				
T4	87 (24.5%)	37 (24.2%)					
Other	268 (75.5%)	116 (75.8%)					

Data are presented as n (%), except where noted. * *p* value $$ <$$ 0.05.

Abbreviations: LVI: lymphovascular invasion; PI, pleural invasion; EGFR: epidermal growth factor receptor; KRAS: kirsten rat sarcoma viral oncogene; ALK: anaplastic lymphoma receptor tyrosine kinase; TP53: tumor protein p53; PIK3CA: p110α catalytic subunit of PI3K; ROS1, c-ROS proto-oncogene 1; PD-1: programmed death 1 receptor; PD-L1: programmed death ligand 1

### Deep learning radiomics architecture

**Fig. 1:**
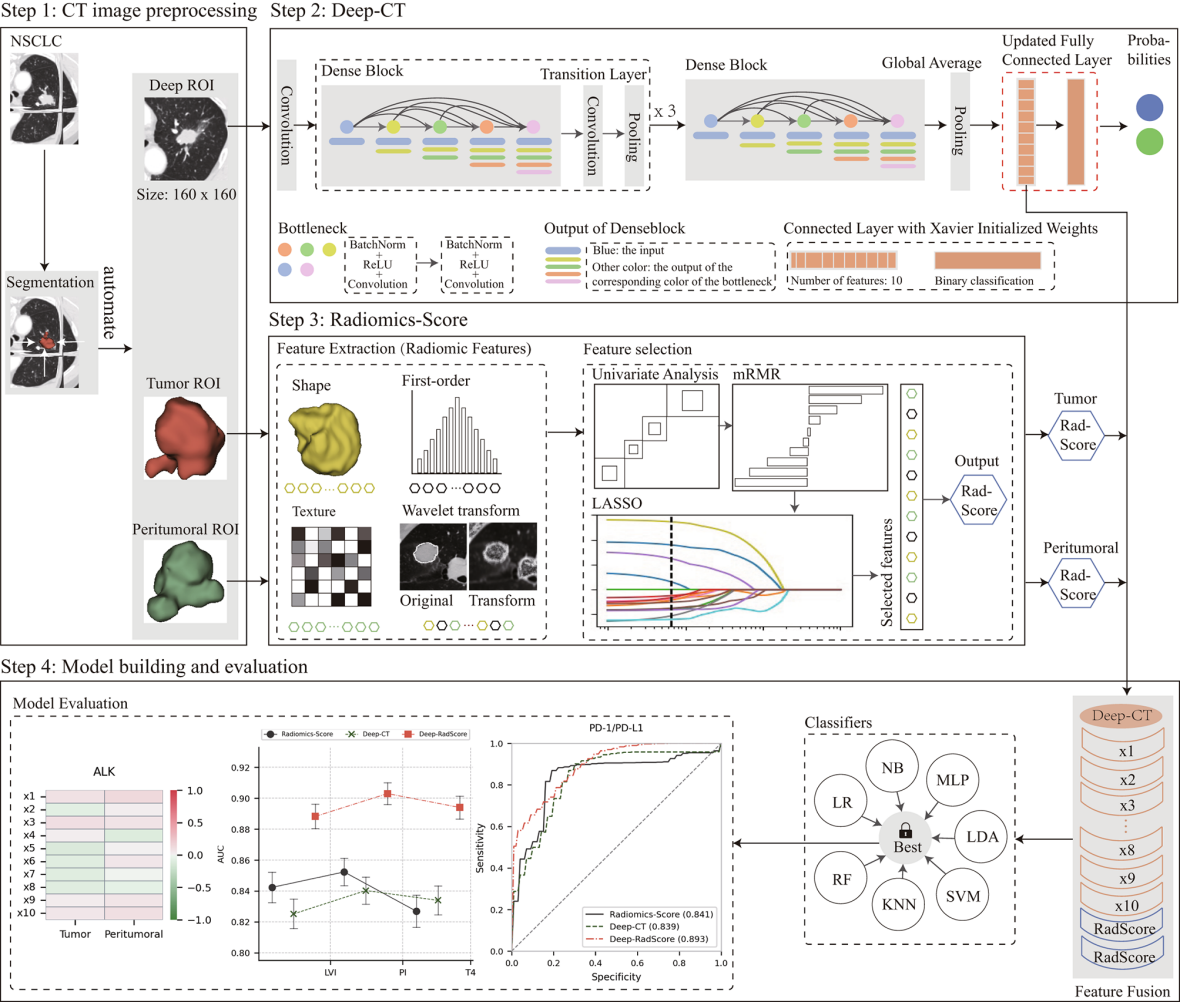
Overall study design. Step 1: CT image preprocessing, three ROIs are utilized as input images. Step 2: CT deep learning model schematic. The pre-trained DenseNet121 is employed as the backbone network to encode input images into network features. Step 3: Radiomics model workflow. Step 4: Development and integration details of the deep learning radiomics model. ROI: region of interest

The overall approach devised is summarized in Fig. 1. We performed clinical information gathering, pathology testing, and CT image collation to train and validate CT-derived radiomic features and deep network features. Subsequently, we employed a multisource feature fusion scheme to predict prognostic risk factors, actionable gene mutation status, and PD-1/PD-L1 expression levels in patients. In brief, we constructed a conventional radiomics model with the radiomic score (RadScore) computed from handcrafted features. After validating our proposed deep network feature-based model as a complementary approach to the conventional radiomics model, we combined these features into a fused model and assessed their clinical performance. Apart from the CT image preprocessing outlined in Step 1, we describe these steps in detail below.

For the CT-based deep learning model (Deep-CT) in Step 2, we utilize DenseNet121, which is pre-trained on the ImageNet dataset, as the backbone network. We employ a training cohort to guide the selection of model hyperparameters, randomly selecting 25% of the training images to form a validation cohort for optimizing the model parameters. The core of our training strategy revolves around supervised learning, where Deep-CT processes all embedded information within the input deep ROIs. The convolutional layer encodes the ROIs and adaptively learns their semantic features, while the fully connected (FC) layer selects relevant features and reduces feature dimensionality. Notably, to maximize the benefits, we redesigned the FC layer, transforming it from 1000 nodes into 2 FC layers with Xavier-initialized weights [25] (in red). In this updated network structure, the output of the penultimate FC layer represents the deep network features. For a more comprehensive understanding of this network, including its inputs, parameters, and detailed structure, please refer to Appendix E2 and Fig. S2. In the third step of our analysis, we develop a radiomics model utilizing conventional radiomic features. This model implements machine learning strategies by training cohorts to predict the outcomes of various molecular alterations. We extract 851 quantitative features for each ROI, encompassing four distinct types: shape, histogram, texture, and filter features. Detailed information regarding feature construction is documented in Appendix E3. Before performing feature selection, all radiomic features were normalized using Z-Score. The normalization parameters computed in the training cohort were applied to adjust the features in the test cohort to match the same mean and variance. The feature selection process adheres to the rigorous methodology outlined in Appendix E4. At the core of our model lies the computation of RadScore for each patient, a crucial metric derived from the selected features. RadScore represents a linear combination of these diverse features, with each feature weighted by its corresponding coefficient. Consequently, our radiomic features provide valuable insights into the various tumor phenotypes, serving as robust predictors for anticipating molecular alterations. The precise formula for RadScore is provided below:

$$ RadScore={f}_{1}*{c}_{1}+{f}_{2}* {c}_{2}+\cdots + {f}_{n-1}*{c}_{n-1}+ {f}_{n}*{c}_{n}$$

where, ${f}_{1}\cdots {f}_{n}$ represents the carefully chosen key features, while ${c}_{1}\cdots {c}_{n}$ denotes the respective coefficients of each feature.

**Table 2:** The performance comparison of the basic network model

		AUC	Accuracy (%)	Sensitivity (%)	Specificity (%)	PPV (%)	NPV (%)
ResNet50	T	0.821 [0.802 0.840]	78.4 [76.4 80.4]	83.5 [80.2 86.8]	65.6 [61.3 69.9]	48.7 [45.6 51.8]	97.5 [96.4 98.6]
	V	0.699 [0.667 0.731]	67.7 [63.4 72.0]	72.3 [64.1 80.5]	61.5 [57.6 65.4]	43.5 [39.9 47.1]	81.7 [80.2 83.2]
	I-T	0.676 [0.656 0.697]	63.5 [60.9 66.1]	71.5 [63.3 79.7]	62.3 [55.6 69.0]	39.2 [37.7 40.7]	80.6 [76.8 84.5]
ResNet101	T	0.763 [0.749 0.777]	74.6 [73.6 75.6]	78.6 [74.6 82.6]	72.9 [71.3 74.5]	65.7 [62.5 68.9]	89.0 [83.9 94.1]
	V	0.649 [0.611 0.668]	62.0 [56.8 67.2 ]	71.6 [61.4 81.8]	59.7 [58.3 61.1]	44.3 [42.0 46.6]	87.8 [83.4 92.3]
	I-T	0.637 [0.604 0.670]	61.4 [55.3 67.5]	71.4 [63.2 80.1]	56.5 [53.0 60.0]	42.5 [37.3 47.7]	82.6 [77.1 88.1]
DenseNet121	T	0.880 [0.866 0.892]	84.5 [83.2 85.8]	92.6 [90.3 94.9]	72.1 [69.3 74.9]	56.9 [53.6 60.2]	98.1 [97.7 98.5]
	V	0.798 [0.765 0.831]	71.2 [66.5 75.9]	93.0 [91.7 94.3]	59.3 [52.2 66.4]	51.3 [44.6 58.0]	90.2 [85.7 94.7]
	I-T	0.792 [0.777 0.807]	68.1 [65.9 70.3]	94.5 [93.4 95.6]	60.3 [56.3 64.3]	48.4 [45.6 51.2]	89.8 [87.5 92.1]
VGG19	T	0.771 [0.758 0.784]	72.8 [67.3 77.7]	87.3 [82.3 92.3]	69.9 [61.0 76.8]	60.8 [54.6 67.0]	91.3 [90.1 92.5]
	V	0.683 [0.664 0.702]	63.5 [61.0 66.0]	79.5 [75.2 83.8]	64.2 [60.0 68.4]	47.5 [46.2 48.8]	86.1 [83.5 88.7]
	I-T	0.679 [0.651 0.707]	62.2 [60.2 64.2]	72.4 [67.3 77.5]	58.1 [55.3 60.9]	45.2 [43.8 46.6]	83.6 [80.6 86.6]
Inception V3	T	0.826 [0.815 0.837]	80.5 [79.3 81.7]	88.5 [84.7 92.3]	73.1 [70.8 75.4]	54.2 [52.6 55.8]	93.4 [91.6 95.2]
	V	0.706 [0.683 0.729]	70.8 [66.7 74.9]	79.3 [76.1 82.5]	64.5 [58.5 70.5]	41.3 [37.9 44.7]	85.6 [82.7 88.5]
	I-T	0.695 [0.657 0.733]	69.3 [66.5 72.1]	75.1 [71.6 78.6]	61.3 [54.5 68.1]	40.8 [38.0 43.6]	82.3 [79.1 85.5]

The values enclosed in square brackets denote 95% confidence intervals.

Abbreviations: T: training cohort (*n* = 267); V: validation cohort (*n* = 88); I-T: independent test cohort (*n* = 153); PPV: positive predict value; NPV: negative predict value

It has been established in Steps 2 and 3 that the ROIs for each patient can be encoded as network features and RadScore. Including an additional valid region may offer even more valuable tumor-related information. Consequently, we extended the framework to accommodate three-source ROI inputs. This extension is implemented through the design of a parallel bimodal structure. Specifically, in Step 1, we employ a network structure consisting of a DenseNet121 backbone network and a customized FC layer. The network features are concatenated and fused by the penultimate FC layer. In Step 2, the inputs consist of both tumor and peritumoral ROIs. Both ROI types undergo an identical radiomics processing pipeline to generate RadScore for the tumor and peritumoral regions. In the combined model, the two RadScore features are directly input into the penultimate FC layer of Deep-CT for fusion, eliminating the need for the offline combination of network features with RadScore.

As depicted in Step 4, the last FC layer is replaced with a combined classifier comprising seven machine learning classification algorithms at the end of the training process. The rationale for employing the combined classifier is elucidated in Appendix E5. Subsequently, employing independent predictors for deep and artificial patterns, we constructed a combined model. This step captured common and complementary valid information from various feature sources and distinct pattern features. Furthermore, it allowed us to determine the optimal prediction model by aggregating predictions from multiple classifiers. A 5-fold cross-validation with ten repetitions was implemented during the training of cohort-based optimization, as detailed in Appendix E6. This methodology identifies the best classifiers for each situation and evaluates performance metrics based on the average area under the curve. After optimization, the resulting predictive models were validated in an independent test cohort to confirm their robustness and generalizability. Additionally, to assess the incremental clinical value of the fusion strategy, we evaluated the disparity between the combined model and the two individual models in predicting the designated clinical endpoints.

**Table 3:** The prediction of prognostic risk factors, gene mutations, and immunoexpression outcomes in the independent test cohort

	Model	Classifier	AUC (95% CI)	Accuracy (%)	Precision (%)	Recall (%)	F1 score (%)	***p***-value
Prognostic risk factors								
LVI	Radiomics-Score	NB	0.842 [0.832–0.852]	69.6	71.2	68.8	79.6	0.043
	Deep-CT	MLP	0.826 [0.816–0.835]	75.1	75.9	74.5	68.8	0.027
	Deep-RadScore	LDA	0.889 [0.881–0.897]	80.4	80.5	84.8	78.5	Baseline
PI	Radiomics-Score	SVM	0.853 [0.844–0.862]	75.4	76.3	69.8	78.0	0.041
	Deep-CT	RF	0.840 [0.831–0.848]	78.7	77.8	79.4	70.6	0.045
	Deep-RadScore	SVM	0.903 [0.896–0.910]	81.6	82.9	84.7	81.5	Baseline
T staging	Radiomics-Score	NB	0.827 [0.817–0.838]	65.6	71.5	72.0	65.6	0.035
	Deep-CT	LDA	0.834 [0.825–0.844]	76.2	78.0	77.9	72.6	0.018
	Deep-RadScore	RF	0.894 [0.886–0.901]	81.5	77.6	84.9	78.6	Baseline
Gene mutations								
EGFR	Radiomics-Score	SVM	0.843 [0.834–0.853]	68.0	70.0	70.7	67.9	0.038
	Deep-CT	NB	0.838 [0.829–0.847]	74.6	76.2	76.2	74.6	0.019
	Deep-RadScore	RF	0.884 [0.876–0.892]	81.9	81.5	83.0	81.5	Baseline
KRAS	Radiomics-Score	MLP	0.841 [0.827–0.855]	78.3	68.7	76.2	69.7	0.039
	Deep-CT	LR	0.830 [0.820–0.841]	69.9	66.7	70.2	66.6	0.017
	Deep-RadScore	LDA	0.896 [0.886–0.906]	80.3	78.1	82.6	78.8	Baseline
ALK	Radiomics-Score	RF	0.823 [0.808–0.838]	72.8	71.4	79.7	74.2	0.002
	Deep-CT	RF	0.821 [0.802–0.840]	68.3	66.5	73.5	62.2	0.0001
	Deep-RadScore	RF	0.884 [0.873–0.895]	83.4	78.6	82.2	78.0	Baseline
TP53	Radiomics-Score	KNN	0.828 [0.817–0.838]	75.8	73.8	75.6	74.2	0.022
	Deep-CT	SVM	0.833 [0.823–0.844]	73.1	73.7	73.3	73.0	0.048
	Deep-RadScore	LDA	0.889 [0.880–0.898]	79.5	80.5	83.2	79.3	Baseline
PIK3CA	Radiomics-Score	KNN	0.839 [0.827–0.851]	81.7	72.3	74.3	74.4	0.045
	Deep-CT	NB	0.824 [0.813–0.835]	73.9	77.8	70.3	70.4	0.0006
	Deep-RadScore	LDA	0.896 [0.886–0.907]	82.6	86.2	79.2	76.8	Baseline
ROS1	Radiomics-Score	MLP	0.827 [0.806–0.849]	73.3	62.5	77.9	61.9	0.018
	Deep-CT	RF	0.832 [0.815–0.849]	72.8	66.4	78.7	66.1	0.015
	Deep-RadScore	NB	0.895 [0.886–0.905]	82.2	78.2	80.4	79.0	Baseline
Immunoexpression								
PD-1/PD-L1	Radiomics-Score	NB	0.841 [0.824–0.858]	79.2	78.7	77.5	77.8	0.024
	Deep-CT	LDA	0.839 [0.823–0.854]	78.4	79.3	79.2	78.4	0.007
	Deep-RadScore	LR	0.893 [0.882–0.905]	82.1	81.5	81.9	81.6	Baseline

*p*-value: DeLong test for the difference in AUC between the Deep-RadScore (baseline) model and the Radiomics-Score/Deep-CT models

Abbreviations: CI: confidence intervals; SVM: support vector machine; KNN: k-nearest neighbors; RF: random forests; NB: naive Bayes classifier; LR: logistic regression; MLP: multilayer perceptron; LDA: linear discriminant analysis

### Correlation and model interpretation

To determine the correlation between the RadScore and the deep network features, we employed the pearson method to calculate correlation coefficients between each pair of features in the fused feature set. Additionally, we computed the average absolute correlation coefficient between the two sets of model features. Furthermore, we utilized the SHAP algorithm [26] to enhance the interpretability of the developed model. This algorithm allowed us to visualize the contribution of each feature to the model’s output and to determine the positive or negative correlation between each feature and the final prediction. Lastly, we employed the Gradient Weighted Class Activation Mapping (Grad-CAM) [27] technique to visualize the Deep-CT network.

**Fig. 2:**
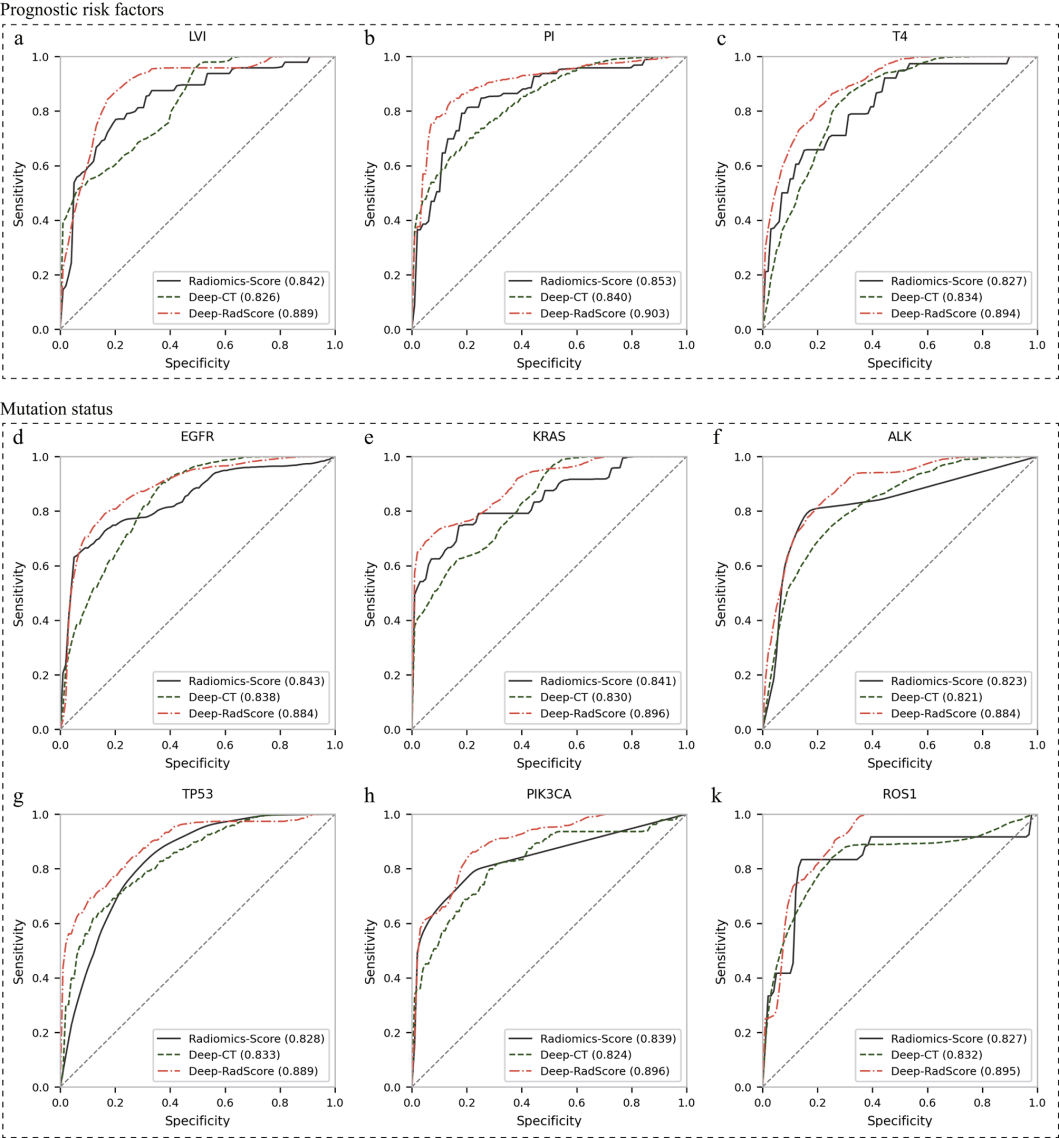
Comparison of receiver operating characteristic (ROC) curves for three models predicting prognostic risk and gene mutations, including LVI **(a)**, PI **(b)**, and T staging **(c)**, as well as for EGFR **(d)**, KRAS **(e)**, ALK **(f)**, TP53 **(g)**, PIK3CA **(h)**, and ROS1 **(i)**. The corresponding area under the curve (AUC) values are indicated in parentheses next to the ROC curves

### Statistical analysis

The Mann-Whitney U test assessed differences in clinicopathologic characteristics among patients in different groups. Receiver operating characteristic (ROC) curves and the area under the ROC curve (AUC) were used to estimate the performance of predictive models. The DeLong test was employed to compare the AUCs and calculate 95% confidence intervals (CI). Other measures, such as accuracy (ACC), precision, recall, and the F1 score, were also assessed. Comparative analyses were conducted using two-sided statistical tests, with p-values less than 0.05 indicative of statistical significance.

**Fig. 3:**
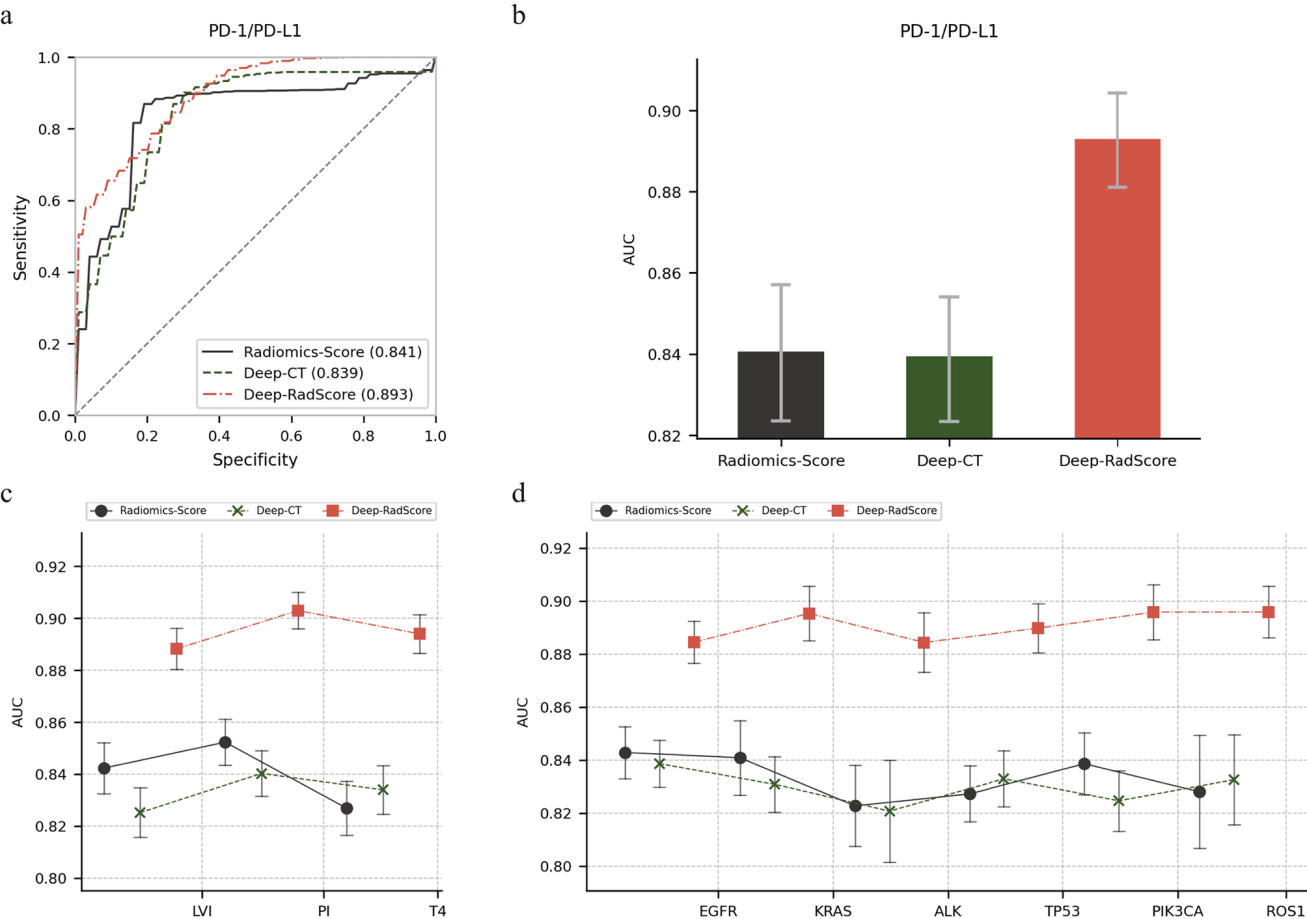
Comparative analysis of receiver operating characteristic (ROC) curves and area under the curve (AUC) distributions. **a)** ROC curves for the three models predicting immune expression levels. **b-d)** AUC distributions for the three models predicting prognostic risk factors, gene mutations, and immune expression levels, respectively

## Results

### Overview of the study design

In the radiomic analysis, challenges related to model robustness and generalization exist. We sought to establish a radiologic treatment decision framework applicable to various molecular alterations. Our analysis included three well-known prognostic risk factors (LVI, PI, and T staging), six gene mutations (EGFR, KRAS, ALK, TP53, PIK3CA, and ROS1), and one immunophenotype (PD-1/PD-L1). To achieve this goal, we employed machine learning and deep neural networks to build a deep learning radiomics model that integrates information from all three tumor regions, thereby reducing the inherent uncertainty associated with a single algorithm and molecule-specific prediction models. Additionally, the choice of classifiers significantly impacts prediction outcomes. We compared the performance of seven different classification algorithms to identify the most suitable model for predicting clinical endpoints.

### Underlying network model selection

In the Deep-CT, the underlying network model serves as a deep feature encoder and plays a crucial role in classification. To select the most suitable deep neural network, we compared the diagnostic performance of ResNet50, ResNet101, DenseNet121, VGG19, and Inception V3 for predicting the LVI status of NSCLC patients. Specific results are presented in Table 2. In the test cohort, DenseNet121 exhibited the best performance with an AUC of 0.792, surpassing the other models (ResNet50: 0.676, ResNet101: 0.637, VGG19: 0.679, and Inception V3: 0.695). Additionally, it achieved the highest negative predictive value, positive predictive value, and specificity, albeit with lower ACC compared to Inception V3 and lower specificity than ResNet50 and Inception V3. Due to its superior AUC performance, we selected DenseNet121 as the backbone network and applied it to other clinical endpoints.

### Imaging features relevant to various molecular alterations

We propose two broad classes of radiological phenotypic features to characterize tumor molecular alterations: bimodal types and spatial heterogeneity. These features are specifically designed to describe the heterogeneity of tumors and the diversity of underlying biological processes. For bimodal types, we employ an efficient representation of the original features through typical radiomics and customized deep neural networks. These methods can quantitatively characterize imaging phenotypes and efficiently represent complex nonlinear patterns. After training, machine learning and deep network encoders will reduce the curse of feature dimensionality while maximally preserving the best representation of the original data. Regarding spatial heterogeneity, we integrate two regions of interest: the primary tumor and its invasive edge. These two regions are complementary and will yield rich radiological phenotypic features to improve the accurate differentiation of tumor characteristics.

### Differentiate the performance of prognostic risk factors

For simplicity and a rigorous, independent evaluation, the performance outcomes presented in this paper are exclusively derived from an independent test cohort. Performance metrics for the training cohort have been documented in the Appendix for reference. In the foundational radiomics model, referred to as the ‘Radiomics-Score,’ two distinct RadScores were computed by selecting critical features from both the tumor ROI and the peritumoral ROI, as extensively elucidated in Appendix E7. The locked Radiomics-Score strategically employed three distinct classifiers to optimize the AUC: NB for LVI and T staging and SVM for PI. Our model achieved commendable AUC values in effectively distinguishing among LVI, PI, and T staging, yielding results of 0.842 (95% CI, [0.832, 0.852]), 0.853 (95% CI, [0.844, 0.862]), and 0.827 (95% CI, [0.817, 0.838]), respectively.

The customized deep network model (Deep-CT) performed similarly to the Radiomics-Score. The model provided better T-staging discrimination with an AUC of 0.834 (higher by 0.07). Furthermore, the ACC, recall, and F1 scores were also the best, albeit with slightly lower precision. For LVI and PI recognition, the Deep-CT did not perform as well as the Radiomics-Score, with AUCs of 0.826 (lower by 0.016) and 0.840 (lower by 0.013), respectively, but obtained higher ACC, precision, and recall. As expected, the Radiomics-Score and Deep-CT captured orthogonal tumor heterogeneity features.

The deep learning radiomics model (Deep-RadScore) performance significantly improved upon integrating enriched quantitative RadScore features with nonlinear deep network features. For LVI, PI, and T staging, Deep-RadScore achieved AUC values of 0.889 (95% CI, [0.881, 0.897]), 0.903 (95% CI, [0.896, 0.910]), and 0.894 (95% CI, [0.886, 0.901]), respectively. These results surpassed those of the Radiomics-Score by 0.047 (p-value = 0.043, DeLong test), 0.05 (p-value = 0.041), and 0.067 (p-value = 0.035) and outperformed the Deep-CT by 0.063 (p-value = 0.027, DeLong test), 0.063 (p-value = 0.045), and 0.06 (p-value = 0.018). Deep-RadScore also demonstrated superior ACC, precision, recall, and F1 scores, except for F1 scores in LVI and precision in T staging. These findings suggest that our fusion strategy, aimed at accommodating diverse pattern features and multiple data sources, is highly effective. The ROC curves and detailed performance metrics for all models predicting LVI, PI, and T staging are shown in Fig. 2a-c; Table 3, respectively.

### Differentiate the performance of alterations in target molecules

As delineated in Table 3, the deep network-based features demonstrated superior performance compared to their handcrafted-based counterparts, yielding AUC values of 0.833 (95% CI, [0.823, 0.844]) and 0.832 (95% CI, [0.815, 0.849]) for discerning TP53 mutation and ROS1 mutation, respectively. Subsequent validation of the deep network-based features revealed a moderate performance, with AUCs of 0.838 (95% CI, [0.829, 0.847]), 0.830 (95% CI, [0.820, 0.841]), 0.821 (95% CI, [0.802, 0.840]), and 0.824 (95% CI, [0.813, 0.835]) for EGFR mutation, KRAS mutation, ALK mutation, and PIK3CA mutation, respectively. Notably, while slightly lower than their corresponding handcrafted-based features, these values exhibited a diminishment of only 0.005, 0.011, 0.011, and 0.012, respectively.

Deep-RadScore, achieved through the amalgamation of two distinct pattern features, demonstrated superior performance compared to Radomics-Score and Deep-CT assessed individually. This performance is quantified by the AUC values and their associated increasing intervals: 0.884 ([0.041, 0.055]) for EGFR, 0.896 ([0.055, 0.066]) for KRAS, 0.884 ([0.061, 0.063]) for ALK, 0.889 ([0.056, 0.061]) for TP53, 0.896 ([0.057, 0.072]) for PIK3CA, and 0.895 ([0.063, 0.068]) for ROS1. An analysis of these AUC values highlights the varying degrees of enhanced predictive capabilities offered by fusion features within the context of different targeting molecules. It’s worth noting that the most modest increase in predictive power was observed in the case of EGFR mutation, which exhibited a 0.041 (p-value = 0.038, DeLong test) improvement compared to the higher single model but remained marginally higher than in the current study. Detailed summaries of predictive performance metrics can be found in Table 3, while Fig. 2d-i presents ROC curves for the three models, providing a comprehensive view of the comparative outcomes.

### Differentiate the performance of immune expression levels

In evaluating PD-1/PD-L1 expression, the most favorable AUC value, amounting to 0.893 (95% CI, [0.882, 0.905]), was attained through the combined utilization of features from the radiomics model and the deep network model. In contrast, employing only the features from either the radiomics or deep network models resulted in AUCs of 0.841 (95% CI, [0.824, 0.858]) and 0.839 (95% CI, [0.823, 0.854]), respectively. Furthermore, the combined model Deep-RadScore consistently demonstrated superior performance in terms of ACC, precision, recall, and F1 scores compared to other models. This remarkable consistency in performance underscores the exceptional predictive capabilities of Deep-RadScore concerning immune molecular phenotypes, as depicted in Fig. 3a; Table 3. Furthermore, within Fig. 3b-d, the AUC distributions provide additional clarity regarding the differences in the performance of the three models concerning the identification of prognostic risk factors, gene mutations, and immune expression levels.

**Fig. 4:**
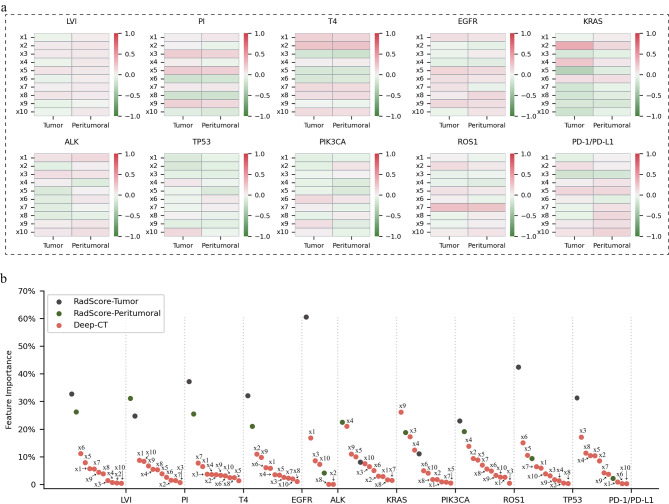
Correlation and significance analysis of different imaging features. **a.** Correlation heatmap of deep network features and radiomic score (RadScore) features for various molecular alterations. **b.** Distribution of the contributions of deep network features and RadScore features in predicting various molecular alterations. ‘Tumor’ represents the RadScore for the tumor region and ‘Peritumoral’ for the peritumoral region

As expected, the fusion features, derived from radiomics and a custom-designed deep network, exhibited significant correlations with alterations in various molecular profiles. This finding underscores their potential utility as a predictive tool for characterizing tumor molecular signatures. Furthermore, this investigation confirms the presence of potential synergistic effects between the proposed conventional handcrafted features and the tailored deep network features.

### Relevance and interpretability of deep-radScore

The assessment of their correlation served to understand the potential synergistic interplay between RadScore and deep network features. The analysis of absolute values and mean absolute values $\pm$ standard deviations of the correlation coefficients for various tumor molecular features produced the following results: $\le$ 0.418 (0.252 $\pm$ 0.114) for LVI, $\le$ 0.679 (0.402 $\pm$ 0.188) for PI, and $\le$ 0.661 (0.375 $\pm$ 0.260) for T staging; $\le$ 0.467 (0.152 $\pm$ 0.144) for EGFR, $\le$ 0.540 (0.377 $\pm$ 0.100) for KRAS, $\le$ 0.316 (0.170 $\pm$ 0.088) for ALK, $\le$ 0.336 (0.232 $\pm$ 0.128) for TP53, $\le$ 0.263 (0.154 $\pm$ 0.073) for PIK3CA, and $\le$ 0.103 (0.052 $\pm$ 0.043) for ROS1; and $\le$ 0.246 (0.113 $\pm$ 0.068) for PD-1/PD-L1. These findings show weak correlations, suggesting that the deep network features offer unique information beyond traditional radiomic features.

When visualizing the contributions of individual features to the final prediction of Deep-RadScore, a discernible correlation emerged between the deep network features and various tumor molecular alterations. Notably, RadScore also exhibited a consistent correlation pattern. In summary, Fig. 4a presents a heatmap illustrating the correlation between RadScore and the deep network features, while Fig. 4b delineates the contribution ratio of each feature. Furthermore, the analysis of each feature’s positive or negative correlation with distinct tumor molecular alterations is detailed in Fig. S3-S5.

To highlight the learning focus of the deep network on the classification target, we employed Grad-CAM to generate a rough localization heat map. As illustrated in Fig. S6, the last convolutional layer of this architecture is transparent to the distinction of LVI status. We found the significant relevance of the tumor and its surrounding context within the CT scans for the accurate differentiation of LVI status by Deep-RadScore. This phenomenon enhances the visual interpretation and validation of the model.

## Discussion

In this comprehensive study, we developed a deep learning radiomics framework utilizing multiple populations of patient data from three institutions. Our primary objective was to achieve precise predictions regarding tumor heterogeneity, response to targeted therapy, and response to immunotherapy in NSCLC. Our proposed radiological phenotypic features demonstrated exceptional discriminatory power, effectively stratifying clinically and therapeutically significant molecular attributes. These encompassed crucial radiological prognostic risk factors, including LVI, PI, and T staging. We also assessed gene mutation statuses thoroughly, encompassing EGFR, KRAS, ALK, TP53, PIK3CA, and ROS1, and evaluated PD-1/PD-L1 expression levels. Notably, we identified a synergy between our customized deep network features and existing radiomic features. These insights culminated in developing a fusion model that seamlessly integrated multiple data sources to optimize predictive efficacy. In summary, our proof-of-concept framework underscores the critical role of deep learning-derived network features extracted from CT images. These features provide complementary insights to established radiological imaging biomarkers, bringing us significantly closer to realizing personalized treatment strategies for NSCLC.

Previous NSCLC radiomic analyses have primarily focused on establishing non-invasive biomarkers for specific clinical endpoints, including diagnosis [28, 29], treatment response [30, 31], and prognostic assessment [32, 33]. While these studies have confirmed that radiomic features can capture relevant molecular biomarkers, their clinical utility could be improved by the fundamental limitation that these imaging features are only modest predictors of specific molecular characteristics. They cannot yet be considered complete substitutes for molecular biomarkers. Moreover, it’s crucial to note that the molecular characteristics of a tumor may dynamically change during treatment [34]. As a result, the clinical value of imaging alternatives for a single or a few molecular features still requires improvement. To overcome this limitation, we aim to identify radiological phenotypes encompassing a broader spectrum of molecular features, aligning them more closely with clinical reality. By employing a deep learning radiomics framework that can longitudinally predict diverse tumor molecular features, we further enhance the practical value of this approach in guiding clinical treatment decisions.

In contrast to conventional radiomics studies, which primarily focus on characterizing the tumor region, we assume that both the tumor region and peritumoral regions contain phenotypic patterns reflecting the underlying biological processes of cancer. The peritumoral region is automatically derived from the segmented 3D tumor region using an in-house algorithm defined from a ‘geometric perspective.’ This method is easily implementable and applicable to various imaging modalities and cancer types. Recent evidence supports the plausibility of this hypothesis, given the clinical relevance of these two regions [35–39]. Moreover, traditional radiomics studies often rely on handcrafted radiomic features [40–44], making them susceptible to uncertainties related to robustness and generalization. This susceptibility remains a significant impediment to model deployment. To address this challenge, we utilize a customized DenseNet-121 architecture to extract fundamentally distinct and potentially complementary deep network features, thereby fully leveraging the capabilities of CT imaging. By combining these network features, which are not predefined within the framework of multiple data sources, we effectively mitigate modeling uncertainties and provide complementary value. This is evident in the strong performance of our fusion model in an independent test cohort.

Radiomics and deep learning, the two primary approaches to radiomic analysis, form the foundation of this research field [13, 14, 45]. Our fusion of these two imaging features introduces a new dimension to radiomic analysis, differentiating it from studies solely based on either modality. Cui et al. [46] reported that combining deep learning and handcrafted features outperformed single imaging features in predicting responses to neoadjuvant chemotherapy in advanced gastric cancer. However, their approach constructed imaging features solely from 2D slices of CT images, neglecting the entire tumor. Miao et al. [18] found that combining deep learning with clinicopathological features improved breast cancer prognosis compared to a single modality. Nevertheless, their deep network features were extracted from entire single slices of CT images without region-specific focus. Our study addresses these limitations and bridges a gap in radiomic analysis by fusing radiomic features of 3D regions with region-specific deep network features for analysis. It’s essential to note that the deep network features are extracted from the largest slice of the entire tumor and may still not fully represent the entire tumor, which could be a major reason for the poor performance of the Deep-CT. This potential limitation underscores the need for further investigation into 3D analysis of the whole tumor using deep networks.

Another advantage of the developed architecture is its support for prognostic risk assessment metrics, key established predictive biomarkers, and immune biomarkers consistent with current NCCN guidelines [4] for NSCLC. To the best of our knowledge, our study is the first radiomics study to simultaneously identify these molecular features relevant to NSCLC treatment. We collected multicenter data from three institutions, utilizing different CT scanners and imaging protocols, which increases confidence in the deep learning radiomics model. Additionally, we observed that the newly acquired deep learning network features demonstrated similar or even higher predictive power than the validated radiomic features. The results indicate that Deep-CT shows a decrease in AUC ranging from − 0.016 to -0.002 in supported molecular characterization predictions compared to Radiomics-Score. However, it demonstrates an increase in predicting T staging (0.007), TP53 mutation (0.005), and ROS1 mutation (0.005). Combining the radiomic features with the deep network features jointly provides the best molecular characterization, denoted as Deep-RadScore, outperforming any model constructed with a single modality feature, with AUC increases ranging from 0.039 to 0.070. We believe that traditional radiomic features only capture specific aspects of tumor molecules. Patients with similar radiomic features can exhibit different molecular characteristics, which might explain why Radomics-Score performs poorly across different patient distributions. Deep network architectures can mine high-dimensional nonlinear patterns of imaging features, allowing them to synthesize and quantify molecular features of tumors, thereby significantly improving discriminative power.

To enhance the interpretability of Deep-RadScore, we employ two strategies. Firstly, we visualize the correlation analysis between different imaging features, demonstrating the contribution of each feature to the prediction results and its positive or negative influence. As highlighted in Fig. 4a, the low pearson correlation coefficient and weak linear correlation between the radiomic features and the deep network features suggest that these two imaging features are complementary rather than redundant. Consequently, their fused features result in more substantial predictive power. Secondly, despite the superior performance achieved by Deep-RadScore, the ‘black-box’ nature of deep learning models presents significant challenges for interpretation. To mitigate this effect, we introduce intuitive roles (as shown in Fig. 4b) and effects (detailed in Fig. S3-S5) for each feature within Deep-RadScore, facilitating the visual interpretation of the features encoded within the deep network. This visual interpretation of the deep network is further supplemented by introducing predicted salient regions generated by Grad-CAM.

This study presents several noteworthy limitations. First, despite including different patient populations from various institutions, inherent data bias is inevitable due to its retrospective nature and variations in patient distribution. Therefore, further well-designed prospective studies in large multicenter cohorts are needed to validate the generalizability and clinical applicability of this deep learning radiomics architecture. Second, available data for rare cases such as ALK mutation (*n* = 27) and ROS1 mutation (*n* = 26) are still limited due to epidemiologic constraints. Additionally, aligning CD8 cell infiltration subgroups with PD-1/PD-L1 expression levels is not sufficiently rigorous, as high CD8 cell infiltration only indicates preferential expression of PD-1/PD-L1. The predictive value of Deep-RadScore for different tumor molecular features should be further validated by comparison with a complete cohort of NSCLC patients with homogeneity. Third, our focus on molecular biomarkers for NSCLC treatment aimed to discover and validate the corresponding imaging biomarkers. However, these biomarkers offer limited prediction of prognostic risk, response to targeted therapy, and response to immunotherapy, with assessment of prognostic outcomes notably absent. Future work should incorporate patient prognostic data for outcome assessment to better inform first-line treatment decisions. Finally, as a result of concept generation, we observed correlations between radiomic features and deep network features with prognostic risk factors, gene mutations, and immunophenotypes. Nevertheless, the biological significance of deep network features remains to be further elucidated. Future studies should seek to understand the expression mechanisms of imaging biomarkers, establish radiogenomic features with causal relationships, and unveil the underlying biology driven by deep learning.

## Conclusion

In summary, we have developed and validated a deep learning radiomics framework based on CT imaging for therapeutic decision support in NSCLC patients. The proposed Deep-RadScore combines handcrafted radiomic features with deep learning encoded network features and offers valuable evidence for predicting three prognostic risk factors, six gene mutations, and one immune molecule. This provides additional value for the precision treatment of patients. However, it’s important to note that these findings require further confirmation through future prospective studies to refine and assess their clinical utility.

### Electronic supplementary material

Below is the link to the electronic supplementary material.

Supplementary Material 1

## Data Availability

Due to patient privacy, the data related to patients is not available for public access. However, it can be obtained from the corresponding author upon reasonable request, subject to approval by the institutional review board of the enrolled centers. The proposed original code, model objects, and test data are available on GitHub. (https://github.com/zxpPro/DeepRadScore_NSCLC).

## Abbreviations

NSCLC: non-small cell lung cancer
LVI: lymphovascular invasion
PI: Pleural invasion
ROI: region of interest
TPS: Tumor Proportion Score
RadScore: radiomic score
Deep-CT: CT-based deep learning model
Deep-RadScore: deep learning radiomics model
FC: fully connected
Grad-CAM: Gradient Weighted Class Activation Mapping
ROC: receiver operating characteristic
AUC: area under the curve
ACC: accuracy
SVM: support vector machine
KNN: k-nearest neighbors
RF: random forests
NB: naive Bayes classifier
LR: logistic regression
MLP: multilayer perceptron
LDA: linear discriminant analysis
CI: confidence intervals
