# Supplementary material for: Exploring non-invasive precision treatment in non-small cell lung cancer patients through deep learning radiomics across imaging features and molecular phenotypes
Source: Biomark Res. 2024 Jan 25;12:12. doi: 10.1186/s40364-024-00561-5 (PMC10809593; doi:10.1186/s40364-024-00561-5)
Supplement: Supplementary file 1 — Supplementary Material 1 [file 40364_2024_561_MOESM1_ESM.docx]

1. Patient recruitment programs

The First Affiliated Hospital of Gannan Medical University conducted strict recruitment criteria (Cohort I). The inclusion criteria were as follows: a, histologically diagnosed non-small cell lung cancer (NSCLC); b, patients with complete clinicopathologic data; c, patients who underwent chest CT within one month before treatment; and d, molecular assays or immunohistochemistry tests were performed, resulting in the presence of EGFR, KRAS, ALK, TP53, PIK3CA and ROS1 status, or PD-1/PD-L1 expression levels. The exclusion criteria were as follows: a, unsatisfactory quality of CT images; b, lack of complete clinicopathological data; c, patients with preoperative antitumor therapy; and d, patients with other malignancies.

Cohort II was derived from the publicly accessible NSCLC Radiogenomics dataset and the selection of eligible patients adhered to analogous inclusion and exclusion criteria. Detailed documentation of the patient recruitment process is presented in Fig. S1.

1. Backbone network

**Network model input**: Before training the DenseNet121 network, rectangular regions of interest (ROIs) are cropped from manually delineated tumor regions, resized to 160 x 160 pixels, and normalized. These preprocessed ROIs are consistently used during training for parameter refinement via backpropagation. Input images also undergo stochastic transformations, including random horizontal and vertical flipping, to mitigate overfitting.

**Network model parameters**: The Deep-CT is built upon DenseNet121, inheriting all convolutional layers. It comprises a 160x160x3 input layer, a 7x7 convolutional layer, three Dense Blocks with Transition Layers, one additional Dense Block, and a final 7x7 global average pooling layer. The original 1,000-neuron Fully Connected Layer (FC) in DenseNet121 is replaced with a 10-neuron FC layer initialized using the Xavier method. The model employs BCEWithLogitsLoss for classification, with additional settings: learning rate of 1e-6, Adam optimizer, batch size of 32, max 1000 iterations, and step size settings of 200 with a gamma value of 0.5. Refer to Fig. S2 for a detailed network structure.

1. Extraction of handcrafted features

The widely applied Python version of the "PyRadiomics" (v3.0.1) package was utilized to extract radiomic characteristics from each of the two ROIs (tumor and peritumoral). Wavelet analysis with three-dimensional low (L) and high (H) spatial frequency filters was applied to each ROI to obtain additional information about the frequency and location of the image. Specifically, each pattern contains one primitive signature group and eight wavelet signature groups. The composition of the primitive characteristics groups is as follows:

Fourteen shape-based features describe the size and shape of the ROI.

Eighteen first-order statistical features reflect the grayscale frequency distribution of pixel or voxel intensities.

Twenty-four gray level co-occurrence matrix (GLCM) features describe the intensity relationships between voxel pairs (second-order gray levels).

Sixteen gray level run length matrix (GLRLM) features are defined as the length of consecutive voxels with the same gray value in the specified direction in the image.

Sixteen gray level size zone matrix (GLSZM) features are defined as the number of adjacent voxels with the same gray level in the image.

Five neighboring gray level difference matrix (NGTDM) features refer to the different levels of each voxel in the image with neighboring voxels within a preset distance, i.e., the sum of the differences of the average gray level values.

Fourteen gray level dependence matrix (GLDM) features refer to the number of connected voxels in the image within a preset distance, reflecting the dependence of gray levels.

Each wavelet feature group includes all the previously mentioned features, totaling 93, except for shape features. This exclusion is grounded in the principle that features unrelated to gray values are not computed in the wavelet-transformed image. Consequently, the composition of the incorporated 851 features is as follows:

Original feature group: $14 +18 +24+16+16+5+14=107$.

Wavelet feature group: $93\times8=744$.

Overall teature: $107+744\equiv851$.

In addition, the parameters of PyRadiomics are as follows: imageType: original and wavelet; interpolator: sitkBSpline; binWidth: 25; resampledPixelSpacing: determined by experiments.

1. Selection of handcrafted features

**Step 1: Univariate analysis.** First, those characters with approximately constant values were excluded. Second, the Mann-Whitney U test and Pearson linear correlation coefficient were utilized to measure the differences in the distribution of features and to calculate the correlation between characteristics, respectively. Those traits with p-values less than 0.05 and correlation coefficients less than 0.75 were retained.

**Step 2: Multivariate minimum redundancy maximum relevance (mRMR).** With the mRMR algorithm, we look for characteristics with maximum relevance to the final output and minimum relevance to other characters in the subset of features filtered in the previous step. The Max-Relevance constraint seeks variables with maximum mutual information between individual traits and categorical labels, while the Min-Redundancy constraint identifies the relevant variables with high dependency.

**Step 3: Embedded least absolute shrinkage and selection operator (LASSO).** The abovementioned univariate and multivariate filtering methods are performed without considering the model. The LASSO algorithm embeds the feature selection into the model construction process and utilizes the model evaluation results for character evaluation. The coefficients of insignificant characteristics are zero with the LASSO method, and the remaining traits with non-zero coefficients are considered the best. These eligible signatures are finally selected for further model building.

1. Introduction to the combined classifiers used

Following best machine learning (ML) practices, we employed a combined classifier comprising seven commonly used ML algorithms. These include logistic regression (LR) as a linear model, linear discriminant analysis (LDA) for discriminant analysis, random forest (RF) as an ensemble method, k-nearest neighbors (KNN) for instance-based modeling, naive Bayes (NB) as a probabilistic classifier, linear support vector machine (SVM), and multi-layer perceptron (MLP) as a neural network method.

The motivation for using multiple classifiers is to enhance result robustness, as prior studies have shown that choosing the suitable algorithm plays an important role in the classification results. For example, when predicting gene mutation status, certain classifiers like RF and SVM have exhibited strong performance in cases like EGFR and KRAS [1-4], while LR has proven effective in BAP1 predictions [5]. Arefan et al. [6] conducted a radiomics study to predict axillary lymph node status in DCE-MRI images using LDA. Liao et al. [7] applied the MLP model and four distinct radiomics methods to forecast overall survival in patients with lung cancer brain metastases after Gamma Knife radiosurgery. The area under the curve (AUC) for survival status at 3, 6, 12, and 24 months was 0.82, 0.80, 0.84, and 0.92, respectively. Ortiz-Ramon et al. [8] compared the performances of five distinct prediction models in an MR image-based radiomics approach. They found that the NB classifier achieved the best results distinguishing between lung cancer and melanoma, consistently achieving a mean AUC exceeding 0.9 in all cases.

When combining various classifiers, Avard et al. [9] developed a framework comprising ten different ML algorithms for identifying myocardial infarction patients. They found that LR and SVM exhibited the best performance for radiomic analysis. Similarly, Alhussaini et al. [10] used two feature types and five ML classifiers to distinguish between benign and malignant renal tumor cells in CT radiomics. Their study yielded the most accurate and reliable ML method from various feature types and classifier combinations. In another radiomics study by Hu et al. [11], the best classifier, which integrated intratumor and peritumoral features, was used to assess the model's predictive performance for pathological complete response after neoadjuvant chemotherapy in esophageal squamous cell carcinoma patients. They reported an AUC of 0.852 for the combined model on the test set. These findings underscore that different feature types and classifier combinations can be customized to suit specific classification tasks. Recognizing the absence of a one-size-fits-all model in ML, we explore multiple feature group types, feature selectors, and classifiers in our study. Our objective is to develop a high-performance and robust model capable of predicting three well-known prognostic risk factors (LVI, PI, and T staging), six gene mutations (EGFR, KRAS, ALK, TP53, PIK3CA, and ROS1), and one immunophenotype (PD-1/PD-L1).

1. Five-fold cross validation

In the context of 5-fold cross-validation, each pairing of pattern features and classifiers produces five areas under the receiver operating characteristic (ROC) curve (AUC). This cross-validation procedure iterates ten times, and the resulting 50 (10x5) AUCs are averaged to characterize the predictive accuracy of specific combinations of pattern features and classifiers. The combination exhibiting the highest average AUC across models is identified as optimal.

Subsequently, the training/validation process that yields the highest AUC is selected to formulate the final models: Radiomics-Score, Deep-CT, and Deep-RadScore. The average of these 50 results serves as a comprehensive performance metric for the predictive model within the training cohort (Table S11).

1. Selected handcrafted features and RadScores
2. LVI status

The formula for RadScores is as follows:

- RadScore (3D) = f_3D_1 *_ c___3D_1 +_ f_3D_2 *_ c___3D_2 +_ f_3D_3 *_ c___3D_3 +_ f_3D_4 *_ c___3D_4 +_ f_3D_5 *_ c___3D_5 +_ f_3D_6 *_ c___3D_6 +_ f_3D_7 *_ c___3D_7 +_ f_3D_8 *_ c___3D_8 +_ f_3D_9 *_ c___3D_9 +_ f_3D_10 *_ c___3D_10 +_ f_3D_11 *_ c___3D_11 +_ f_3D_12 *_ c___3D_12 +_ f_3D_13 *_ c___3D_13 +_ f_3D_14 *_ c___3D_14_
- RadScore (peritumoral) = f_Per_1 *_ c___Per_1 +_ f_Per_2 *_ c___Per_2 +_ f_Per_3 *_ c___Per_3 +_ f_Per_4 *_ c___Per_4 +_ f_Per_5 *_ c___Per_5 +_ f_Per_6 *_ c___Per_6 +_ f_Per_7 *_ c___Per_7 +_ f_Per_8 *_ c___Per_8 +_ f_Per_9 *_ c___Per_9_

, where the abbreviations in the formula are shown in Table S1.

1. The best features selected and their respective RadScore calculation formulas for predicting LVI status.

|  | Radiomic feature | Abbreviation | Coefficient | Abbreviation |
| --- | --- | --- | --- | --- |
| 3D ROI | | | | |
|  | original_gldm_SmallDependenceLowGrayLevelEmphasis | f_3D_1_ | -0.115706116 | c_3D_1_ |
|  | wavelet-LHH_glszm_GrayLevelNonUniformityNormalized | f_3D_2_ | 0.003103356 | c_3D_2_ |
|  | wavelet-HLL_gldm_LargeDependenceHighGrayLevelEmphasis | f_3D_3_ | -0.052990372 | c_3D_3_ |
|  | wavelet-HHH_glszm_LargeAreaHighGrayLevelEmphasis | f_3D_4_ | -0.047697815 | c_3D_4_ |
|  | wavelet-HHH_glszm_HighGrayLevelZoneEmphasis | f_3D_5_ | -0.013602256 | c_3D_5_ |
|  | original_glcm_Imc2 | f_3D_6_ | -0.043452711 | c_3D_6_ |
|  | wavelet-HLL_glcm_MCC | f_3D_7_ | 0.033630533 | c_3D_7_ |
|  | wavelet-LLL_glcm_JointEnergy | f_3D_8_ | 0.047434537 | c_3D_8_ |
|  | wavelet-LLH_glcm_Imc2 | f_3D_9_ | 0.067381595 | c_3D_9_ |
|  | wavelet-LLH_glcm_ClusterShade | f_3D_10_ | 0.05793583 | c_3D_10_ |
|  | wavelet-LLH_glcm_Correlation | f_3D_11_ | -0.012338856 | c_3D_11_ |
|  | wavelet-HHH_glcm_Imc1 | f_3D_12_ | 0.030395293 | c_3D_12_ |
|  | wavelet-LHL_glcm_Autocorrelation | f_3D_13_ | -0.017825681 | c_3D_13_ |
|  | original_shape_Sphericity | f_3D_14_ | -0.045619367 | c_3D_14_ |
| Peritumoral ROI | | | | |
|  | original_shape_Sphericity | f_Per_1_ | -0.063970606 | c_Per_1_ |
|  | wavelet-HLL_firstorder_Skewness | f_Per_2_ | 0.05788682 | c_Per_2_ |
|  | wavelet-HLL_glszm_LowGrayLevelZoneEmphasis | f_Per_3_ | -0.030766907 | c_Per_3_ |
|  | wavelet-LLL_gldm_LargeDependenceLowGrayLevelEmphasis | f_Per_4_ | -0.015112351 | c_Per_4_ |
|  | wavelet-LLH_gldm_DependenceEntropy | f_Per_5_ | 0.107077323 | c_Per_5_ |
|  | wavelet-LLL_ngtdm_Strength | f_Per_6_ | -0.023050098 | c_Per_6_ |
|  | wavelet-HHL_glcm_Imc1 | f_Per_7_ | 0.001119378 | c_Per_7_ |
|  | wavelet-HLH_glcm_Imc2 | f_Per_8_ | -0.037471664 | c_Per_8_ |
|  | wavelet-LLL_firstorder_10Percentile | f_Per_9_ | 0.115654743 | c_Per_9_ |

1. PI status

The formula for RadScores is as follows:

- RadScore (3D) = f_3D_1 *_ c___3D_1 +_ f_3D_2 *_ c___3D_2 +_ f_3D_3 *_ c___3D_3 +_ f_3D_4 *_ c___3D_4 +_ f_3D_5 *_ c___3D_5 +_ f_3D_6 *_ c___3D_6 +_ f_3D_7 *_ c___3D_7 +_ f_3D_8 *_ c___3D_8 +_ f_3D_9 *_ c___3D_9 +_ f_3D_10 *_ c___3D_10 +_ f_3D_11 *_ c___3D_11_
- RadScore (peritumoral) = f_Per_1 *_ c___Per_1 +_ f_Per_2 *_ c___Per_2 +_ f_Per_3 *_ c___Per_3 +_ f_Per_4 *_ c___Per_4 +_ f_Per_5 *_ c___Per_5 +_ f_Per_6 *_ c___Per_6 +_ f_Per_7 *_ c___Per_7 +_ f_Per_8 *_ c___Per_8 +_ f_Per_9 *_ c___Per_9 +_ f_Per_10 *_ c___Per_10 +_ f_Per_11 *_ c___Per_11 +_ f_Per_12 *_ c___Per_12 +_ f_Per_13 *_ c___Per_13 +_ f_Per_14 *_ c___Per_14 +_ f_Per_15 *_ c___Per_15 +_ f_Per_16 *_ c___Per_16 +_ f_Per_17 *_ c___Per_17 +_ f_Per_18 *_ c___Per_18_

1. The best features selected and their respective RadScore calculation formulas for predicting PI status.

|  | Radiomic feature | Abbreviation | Coefficient | Abbreviation |
| --- | --- | --- | --- | --- |
| 3D ROI | | | | |
|  | wavelet-LLL_glcm_InverseVariance | f_3D_1_ | 0.267271389 | c_3D_1_ |
|  | wavelet-HLH_glszm_LargeAreaEmphasis | f_3D_2_ | -0.007735556 | c_3D_2_ |
|  | wavelet-HHH_glcm_Idmn | f_3D_3_ | 0.091147144 | c_3D_3_ |
|  | original_glszm_SmallAreaLowGrayLevelEmphasis | f_3D_4_ | 0.034035607 | c_3D_4_ |
|  | original_ngtdm_Strength | f_3D_5_ | -0.034672621 | c_3D_5_ |
|  | wavelet-LLL_firstorder_Minimum | f_3D_6_ | 0.012115207 | c_3D_6_ |
|  | wavelet-HHH_glszm_HighGrayLevelZoneEmphasis | f_3D_7_ | 0.063616047 | c_3D_7_ |
|  | original_glcm_Imc2 | f_3D_8_ | 0.057241757 | c_3D_8_ |
|  | wavelet-LLH_glcm_MCC | f_3D_9_ | 0.017211935 | c_3D_9_ |
|  | wavelet-HLL_gldm_LargeDependenceHighGrayLevelEmphasis | f_3D_10_ | -0.020744339 | c_3D_10_ |
|  | wavelet-LHL_firstorder_Kurtosis | f_3D_11_ | 0.066095497 | c_3D_11_ |
| Peritumoral ROI | | | | |
|  | original_firstorder_RootMeanSquared | f_Per_1_ | -0.190986217 | c_Per_1_ |
|  | original_shape_MinorAxisLength | f_Per_2_ | 0.075731274 | c_Per_2_ |
|  | wavelet-HLH_ngtdm_Strength | f_Per_3_ | 0.021025706 | c_Per_3_ |
|  | original_gldm_DependenceEntropy | f_Per_4_ | 0.123650932 | c_Per_4_ |
|  | wavelet-HLH_glcm_Idn | f_Per_5_ | 0.007071233 | c_Per_5_ |
|  | wavelet-HHH_glcm_Idn | f_Per_6_ | 0.023122251 | c_Per_6_ |
|  | wavelet-LLL_glszm_LowGrayLevelZoneEmphasis | f_Per_7_ | 0.02464184 | c_Per_7_ |
|  | original_glcm_ClusterShade | f_Per_8_ | -0.041885926 | c_Per_8_ |
|  | wavelet-LLL_ngtdm_Strength | f_Per_9_ | 0.025330935 | c_Per_9_ |
|  | wavelet-HHL_glcm_ClusterShade | f_Per_10_ | 0.026351358 | c_Per_10_ |
|  | wavelet-LLL_glcm_Imc1 | f_Per_11_ | 0.043913923 | c_Per_11_ |
|  | wavelet-LLH_glrlm_LongRunHighGrayLevelEmphasis | f_Per_12_ | 0.003248659 | c_Per_12_ |
|  | wavelet-HHL_firstorder_Kurtosis | f_Per_13_ | 0.005761959 | c_Per_13_ |
|  | original_glcm_Idmn | f_Per_14_ | 0.021728541 | c_Per_14_ |
|  | wavelet-HLL_firstorder_Skewness | f_Pe_r15_ | 0.004012642 | c_Per_15_ |
|  | wavelet-HHH_glszm_SmallAreaEmphasis | f_Per_16_ | -0.06099665 | c_Per_16_ |
|  | wavelet-HLH_glszm_LowGrayLevelZoneEmphasis | f_Per_17_ | -0.03570012 | c_Per_17_ |
|  | wavelet-LHL_glszm_ZoneEntropy | f_Per_18_ | -0.012074736 | c_Per_18_ |

1. T staging

The formula for RadScores is as follows:

- RadScore (3D) = f_3D_1 *_ c___3D_1 +_ f_3D_2 *_ c___3D_2 +_ f_3D_3 *_ c___3D_3 +_ f_3D_4 *_ c___3D_4 +_ f_3D_5 *_ c___3D_5 +_ f_3D_6 *_ c___3D_6 +_ f_3D_7 *_ c___3D_7 +_ f_3D_8 *_ c___3D_8 +_ f_3D_9 *_ c___3D_9 +_ f_3D_10 *_ c___3D_10 +_ f_3D_11 *_ c___3D_11 +_ f_3D_12 *_ c___3D_12 +_ f_3D_13 *_ c___3D_13 +_ f_3D_14 *_ c___3D_14_
- RadScore (peritumoral) = f_Per_1 *_ c___Per_1 +_ f_Per_2 *_ c___Per_2 +_ f_Per_3 *_ c___Per_3 +_ f_Per_4 *_ c___Per_4 +_ f_Per_5 *_ c___Per_5 +_ f_Per_6 *_ c___Per_6 +_ f_Per_7 *_ c___Per_7 +_ f_Per_8 *_ c___Per_8 +_ f_Per_9 *_ c___Per_9 +_ f_Per_10 *_ c___Per_10 +_ f_Per_11 *_ c___Per_11_

1. The best features selected and their respective RadScore calculation formulas for predicting T staging.

|  | Radiomic feature | Abbreviation | Coefficient | Abbreviation |
| --- | --- | --- | --- | --- |
| 3D ROI | | | | |
|  | original_glszm_LargeAreaEmphasis | f_3D_1_ | 0.001655589 | c_3D_1_ |
|  | original_shape_Maximum2DDiameterRow | f_3D_2_ | 0.16699602 | c_3D_2_ |
|  | original_gldm_DependenceVariance | f_3D_3_ | 0.004698509 | c_3D_3_ |
|  | wavelet-LHH_firstorder_Energy | f_3D_4_ | -0.136597365 | c_3D_4_ |
|  | wavelet-LHL_gldm_SmallDependenceHighGrayLevelEmphasis | f_3D_5_ | -0.001628376 | c_3D_5_ |
|  | original_glszm_HighGrayLevelZoneEmphasis | f_3D_6_ | 0.05255513 | c_3D_6_ |
|  | wavelet-LLH_glrlm_RunVariance | f_3D_7_ | 0.105920252 | c_3D_7_ |
|  | wavelet-LLH_glrlm_ShortRunHighGrayLevelEmphasis | f_3D_8_ | -0.03140935 | c_3D_8_ |
|  | wavelet-HHH_glszm_SizeZoneNonUniformityNormalized | f_3D_9_ | 0.020618131 | c_3D_9_ |
|  | wavelet-LLH_firstorder_10Percentile | f_3D_10_ | 0.014406993 | c_3D_10_ |
|  | wavelet-LLL_glcm_Correlation | f_3D_11_ | -0.042813247 | c_3D_11_ |
|  | original_glcm_Imc2 | f_3D_12_ | 0.018812178 | c_3D_12_ |
|  | wavelet-HHH_glcm_Idmn | f_3D_13_ | 0.063936115 | c_3D_13_ |
|  | wavelet-HLL_gldm_LargeDependenceHighGrayLevelEmphasis | f_3D_14_ | -0.017476252 | c_3D_14_ |
| Peritumoral ROI | | | | |
|  | wavelet-HHH_glrlm_GrayLevelNonUniformity | f_Per_1_ | 0.14915067 | c_Per_1_ |
|  | wavelet-HLH_glrlm_GrayLevelVariance | f_Per_2_ | 0.003750082 | c_Per_2_ |
|  | wavelet-LLL_glrlm_LongRunLowGrayLevelEmphasis | f_Per_3_ | -0.105581945 | c_Per_3_ |
|  | wavelet-HLL_firstorder_Maximum | f_Per_4_ | 0.077992983 | c_Per_4_ |
|  | wavelet-HLL_ngtdm_Strength | f_Per_5_ | -0.043976857 | c_Per_5_ |
|  | wavelet-LHL_glcm_Correlation | f_Per_6_ | 0.058154016 | c_Per_6_ |
|  | original_glcm_ClusterShade | f_Per_7_ | -0.042151892 | c_Per_7_ |
|  | wavelet-LLL_glszm_LargeAreaHighGrayLevelEmphasis | f_Per_8_ | -0.056983352 | c_Per_8_ |
|  | wavelet-HLH_firstorder_Energy | f_Per_9_ | -0.07764045 | c_Per_9_ |
|  | wavelet-LLL_firstorder_90Percentile | f_Per_10_ | 0.027480016 | c_Per_10_ |
|  | wavelet-LLL_glcm_Idn | f_Per_11_ | -0.023686268 | c_Per_11_ |

1. EGFR mutation

The formula for RadScores is as follows:

- RadScore (3D) = f_3D_1 *_ c___3D_1 +_ f_3D_2 *_ c___3D_2 +_ f_3D_3 *_ c___3D_3 +_ f_3D_4 *_ c___3D_4 +_ f_3D_5 *_ c___3D_5 +_ f_3D_6 *_ c___3D_6 +_ f_3D_7 *_ c___3D_7 +_ f_3D_8 *_ c___3D_8 +_ f_3D_9 *_ c___3D_9 +_ f_3D_10 *_ c___3D_10 +_ f_3D_11 *_ c___3D_11_
- RadScore (peritumoral) = f_Per_1 *_ c___Per_1 +_ f_Per_2 *_ c___Per_2 +_ f_Per_3 *_ c___Per_3 +_ f_Per_4 *_ c___Per_4 +_ f_Per_5 *_ c___Per_5 +_ f_Per_6 *_ c___Per_6 +_ f_Per_7 *_ c___Per_7 +_ f_Per_8 *_ c___Per_8 +_ f_Per_9 *_ c___Per_9 +_ f_Per_10 *_ c___Per_10 +_ f_Per_11 *_ c___Per_11 +_ f_Per_12 *_ c___Per_12 +_ f_Per_13 *_ c___Per_13 +_ f_Per_14 *_ c___Per_14 +_ f_Per_15 *_ c___Per_15_

1. The best features selected and their respective RadScore calculation formulas for predicting EGFR mutation.

|  | Radiomic feature | Abbreviation | Coefficient | Abbreviation |
| --- | --- | --- | --- | --- |
| 3D ROI | | | | |
|  | wavelet-LHL_glcm_InverseVariance | f_3D_1_ | 0.04309239 | c_3D_1_ |
|  | wavelet-LHH_glcm_SumAverage | f_3D_2_ | -0.071300091 | c_3D_2_ |
|  | wavelet-HLL_glszm_ZoneVariance | f_3D_3_ | -0.018060723 | c_3D_3_ |
|  | wavelet-HHL_firstorder_Skewness | f_3D_4_ | -0.055698752 | c_3D_4_ |
|  | wavelet-HHL_glcm_Imc1 | f_3D_5_ | 0.057816701 | c_3D_5_ |
|  | wavelet-LHL_glszm_SmallAreaLowGrayLevelEmphasis | f_3D_6_ | -0.104639494 | c_3D_6_ |
|  | wavelet-HLL_firstorder_Skewness | f_3D_7_ | 0.171433773 | c_3D_7_ |
|  | wavelet-HHL_glcm_SumAverage | f_3D_8_ | -0.093260265 | c_3D_8_ |
|  | wavelet-HLH_firstorder_Skewness | f_3D_9_ | 0.034768534 | c_3D_9_ |
|  | wavelet-HHH_gldm_LargeDependenceLowGrayLevelEmphasis | f_3D_10_ | -0.068803094 | c_3D_10_ |
|  | original_glszm_HighGrayLevelZoneEmphasis | f_3D_11_ | -0.117471163 | c_3D_11_ |
| Peritumoral ROI | | | | |
|  | wavelet-LLL_firstorder_Minimum | f_Per_1_ | 0.147093899 | c_Per_1_ |
|  | wavelet-HHH_glrlm_RunPercentage | f_Per_2_ | -0.017708348 | c_Per_2_ |
|  | original_glrlm_GrayLevelVariance | f_Per_3_ | -0.013197264 | c_Per_3_ |
|  | original_glrlm_RunLengthNonUniformity | f_Per_4_ | -0.001700592 | c_Per_4_ |
|  | wavelet-LLH_glcm_ClusterTendency | f_Per_5_ | 0.024915478 | c_Per_5_ |
|  | wavelet-HLH_glszm_LowGrayLevelZoneEmphasis | f_Per_6_ | -0.03393596 | c_Per_6_ |
|  | original_glcm_MCC | f_Per_7_ | -0.000779484 | c_Per_7_ |
|  | wavelet-HHH_glszm_SmallAreaEmphasis | f_Per_8_ | -0.023984286 | c_Per_8_ |
|  | original_ngtdm_Contrast | f_Per_9_ | -0.045638756 | c_Per_9_ |
|  | original_glszm_SmallAreaEmphasis | f_Per_10_ | -0.052141938 | c_Per_10_ |
|  | wavelet-LHH_glcm_Imc2 | f_Per_11_ | -0.017358532 | c_Per_11_ |
|  | wavelet-HHH_firstorder_Skewness | f_Per_12_ | -0.040031919 | c_Per_12_ |
|  | original_firstorder_Energy | f_Per_13_ | -0.059442252 | c_Per_13_ |
|  | wavelet-HLH_glcm_Imc2 | f_Per_14_ | -0.06225804 | c_Per_14_ |
|  | wavelet-HLL_gldm_LargeDependenceHighGrayLevelEmphasis | f_Per_15_ | -0.006492633 | c_Per_15_ |

1. KRAS mutation

The formula for RadScores is as follows:

- RadScore (3D) = f_3D_1 *_ c___3D_1 +_ f_3D_2 *_ c___3D_2 +_ f_3D_3 *_ c___3D_3 +_ f_3D_4 *_ c___3D_4 +_ f_3D_5 *_ c___3D_5 +_ f_3D_6 *_ c___3D_6 +_ f_3D_7 *_ c___3D_7 +_ f_3D_8 *_ c___3D_8 +_ f_3D_9 *_ c___3D_9 +_ f_3D_10 *_ c___3D_10 +_ f_3D_11 *_ c___3D_11_
- RadScore (peritumoral) = f_Per_1 *_ c___Per_1 +_ f_Per_2 *_ c___Per_2 +_ f_Per_3 *_ c___Per_3 +_ f_Per_4 *_ c___Per_4 +_ f_Per_5 *_ c___Per_5 +_ f_Per_6 *_ c___Per_6 +_ f_Per_7 *_ c___Per_7 +_ f_Per_8 *_ c___Per_8 +_ f_Per_9 *_ c___Per_9 +_ f_Per_10 *_ c___Per_10 +_ f_Per_11 *_ c___Per_11 +_ f_Per_12 *_ c___Per_12_

1. The best features selected and their respective RadScore calculation formulas for predicting KRAS mutation.

|  | Radiomic feature | Abbreviation | Coefficient | Abbreviation |
| --- | --- | --- | --- | --- |
| 3D ROI | | | | |
|  | wavelet-HHH_glrlm_HighGrayLevelRunEmphasis | f_3D_1_ | 0.095592015 | c_3D_1_ |
|  | wavelet-HHL_glszm_LargeAreaLowGrayLevelEmphasis | f_3D_2_ | 0.010292372 | c_3D_2_ |
|  | original_shape_Maximum2DDiameterSlice | f_3D_3_ | -0.065675876 | c_3D_3_ |
|  | wavelet-LLH_glcm_JointAverage | f_3D_4_ | 0.012433048 | c_3D_4_ |
|  | original_firstorder_Range | f_3D_5_ | 0.016675756 | c_3D_5_ |
|  | original_shape_Elongation | f_3D_6_ | 0.053274123 | c_3D_6_ |
|  | wavelet-HLL_glcm_MCC | f_3D_7_ | -0.06841356 | c_3D_7_ |
|  | wavelet-LLL_firstorder_Energy | f_3D_8_ | -0.016103607 | c_3D_8_ |
|  | wavelet-HHL_gldm_LargeDependenceLowGrayLevelEmphasis | f_3D_9_ | -0.075734441 | c_3D_9_ |
|  | wavelet-HHH_firstorder_Mean | f_3D_10_ | 0.043877857 | c_3D_10_ |
|  | wavelet-LLL_firstorder_90Percentile | f_3D_11_ | -0.035629165 | c_3D_11_ |
| Peritumoral ROI | | | | |
|  | wavelet-HHH_glcm_JointAverage | f_Per_1_ | -0.008429711 | c_Per_1_ |
|  | wavelet-HHL_firstorder_InterquartileRange | f_Per_2_ | 0.120113891 | c_Per_2_ |
|  | wavelet-LLH_glszm_LargeAreaHighGrayLevelEmphasis | f_Per_3_ | 0.044511384 | c_Per_3_ |
|  | original_glcm_ClusterProminence | f_Per_4_ | -0.098511244 | c_Per_4_ |
|  | wavelet-LHL_firstorder_Skewness | f_Per_5_ | -0.056734981 | c_Per_5_ |
|  | wavelet-LLH_firstorder_Range | f_Per_6_ | 0.05410913 | c_Per_6_ |
|  | wavelet-LHL_glcm_Idmn | f_Per_7_ | -0.068291766 | c_Per_7_ |
|  | wavelet-LHL_gldm_DependenceEntropy | f_Per_8_ | 0.041407048 | c_Per_8_ |
|  | original_gldm_LargeDependenceLowGrayLevelEmphasis | f_Per_9_ | 0.009390107 | c_Per_9_ |
|  | original_shape_Elongation | f_Per_10_ | 0.078775317 | c_Per_10_ |
|  | wavelet-HHL_firstorder_Mean | f_Per_11_ | 0.082919306 | c_Per_11_ |
|  | wavelet-LHH_glcm_MCC | f_Per_12_ | 0.093586097 | c_Per_12_ |

1. ALK mutation

The formula for RadScores is as follows:

- RadScore (3D) = f_3D_1 *_ c___3D_1 +_ f_3D_2 *_ c___3D_2 +_ f_3D_3 *_ c___3D_3 +_ f_3D_4 *_ c___3D_4 +_ f_3D_5 *_ c___3D_5 +_ f_3D_6 *_ c___3D_6 +_ f_3D_7 *_ c___3D_7 +_ f_3D_8 *_ c___3D_8 +_ f_3D_9 *_ c___3D_9 +_ f_3D_10 *_ c___3D_10 +_ f_3D_11 *_ c___3D_11 +_ f_3D_12 *_ c___3D_12 +_ f_3D_13 *_ c___3D_13 +_ f_3D_14 *_ c___3D_14_
- RadScore (peritumoral) = f_Per_1 *_ c___Per_1 +_ f_Per_2 *_ c___Per_2 +_ f_Per_3 *_ c___Per_3 +_ f_Per_4 *_ c___Per_4 +_ f_Per_5 *_ c___Per_5 +_ f_Per_6 *_ c___Per_6 +_ f_Per_7 *_ c___Per_7 +_ f_Per_8 *_ c___Per_8 +_ f_Per_9 *_ c___Per_9 +_ f_Per_10 *_ c___Per_10 +_ f_Per_11 *_ c___Per_11 +_ f_Per_12 *_ c___Per_12 +_ f_Per_13 *_ c___Per_13 +_ f_Per_14 *_ c___Per_14 +_ f_Per_15 *_ c___Per_15 +_ f_Per_16 *_ c___Per_16 +_ f_Per_17 *_ c___Per_17_

1. The best features selected and their respective RadScore calculation formulas for predicting ALK mutation.

|  | Radiomic feature | Abbreviation | Coefficient | Abbreviation |
| --- | --- | --- | --- | --- |
| 3D ROI | | | | |
|  | wavelet-HHL_glcm_JointAverage | f_3D_1_ | -0.229144748 | c_3D_1_ |
|  | wavelet-LLL_glcm_MCC | f_3D_2_ | -0.130962162 | c_3D_2_ |
|  | wavelet-LLH_glcm_Imc1 | f_3D_3_ | -0.066783466 | c_3D_3_ |
|  | wavelet-HLH_glcm_Autocorrelation | f_3D_4_ | -0.061400556 | c_3D_4_ |
|  | wavelet-HHH_glszm_SizeZoneNonUniformity | f_3D_5_ | 0.165538992 | c_3D_5_ |
|  | wavelet-HLL_glszm_LargeAreaLowGrayLevelEmphasis | f_3D_6_ | -0.077152962 | c_3D_6_ |
|  | wavelet-HLH_glcm_Imc1 | f_3D_7_ | -0.151905051 | c_3D_7_ |
|  | original_shape_Maximum2DDiameterColumn | f_3D_8_ | -0.074839566 | c_3D_8_ |
|  | wavelet-LHH_firstorder_Skewness | f_3D_9_ | -0.04870674 | c_3D_9_ |
|  | wavelet-HHL_glcm_Correlation | f_3D_10_ | -0.166077244 | c_3D_10_ |
|  | original_glcm_MCC | f_3D_11_ | -0.044649463 | c_3D_11_ |
|  | wavelet-LHH_glrlm_HighGrayLevelRunEmphasis | f_3D_12_ | -0.07379953 | c_3D_12_ |
|  | wavelet-HHL_firstorder_Kurtosis | f_3D_13_ | 0.037606147 | c_3D_13_ |
|  | wavelet-LLL_glszm_ZoneEntropy | f_3D_14_ | 0.156368978 | c_3D_14_ |
| Peritumoral ROI | | | | |
|  | wavelet-HLH_glcm_Autocorrelation | f_Per_1_ | -0.036437125 | c_Per_1_ |
|  | wavelet-LHH_glcm_MaximumProbability | f_Per_2_ | -0.118936478 | c_Per_2_ |
|  | original_glszm_LargeAreaHighGrayLevelEmphasis | f_Per_3_ | -0.01786295 | c_Per_3_ |
|  | original_shape_Elongation | f_Per_4_ | -0.05528068 | c_Per_4_ |
|  | original_gldm_LargeDependenceLowGrayLevelEmphasis | f_Per_5_ | 0.109781813 | c_Per_5_ |
|  | wavelet-LHH_glcm_JointAverage | f_Per_6_ | -0.06096856 | c_Per_6_ |
|  | wavelet-HHL_ngtdm_Strength | f_Per_7_ | -0.029293464 | c_Per_7_ |
|  | wavelet-LLL_firstorder_Minimum | f_Per_8_ | 0.008312872 | c_Per_8_ |
|  | wavelet-HHH_glcm_Idmn | f_Per_9_ | 0.047046117 | c_Per_9_ |
|  | original_shape_SurfaceVolumeRatio | f_Per_10_ | 0.038236261 | c_Per_10_ |
|  | wavelet-HLL_gldm_LargeDependenceLowGrayLevelEmphasis | f_Per_11_ | 0.018499132 | c_Per_11_ |
|  | wavelet-HHH_glcm_Autocorrelation | f_Per_12_ | -0.005922954 | c_Per_12_ |
|  | wavelet-LHL_glcm_Imc2 | f_Per_13_ | 0.043829783 | c_Per_13_ |
|  | wavelet-LHL_gldm_LargeDependenceHighGrayLevelEmphasis | f_Per_14_ | -0.018735337 | c_Per_14_ |
|  | wavelet-LLL_glcm_Imc1 | f_Per_15_ | -0.025216652 | c_Per_15_ |
|  | original_firstorder_Median | f_Per_16_ | 0.181896497 | c_Per_16_ |
|  | wavelet-LHL_firstorder_Maximum | f_Per_17_ | -0.070708878 | c_Per_17_ |

1. TP53 mutation

The formula for RadScores is as follows:

- RadScore (3D) = f_3D_1 *_ c___3D_1 +_ f_3D_2 *_ c___3D_2 +_ f_3D_3 *_ c___3D_3 +_ f_3D_4 *_ c___3D_4 +_ f_3D_5 *_ c___3D_5 +_ f_3D_6 *_ c___3D_6 +_ f_3D_7 *_ c___3D_7 +_ f_3D_8 *_ c___3D_8 +_ f_3D_9 *_ c___3D_9 +_ f_3D_10 *_ c___3D_10 +_ f_3D_11 *_ c___3D_11 +_ f_3D_12 *_ c___3D_12 +_ f_3D_13 *_ c___3D_13 +_ f_3D_14 *_ c___3D_14 +_ f_3D_15 *_ c___3D_15 +_ f_3D_16 *_ c___3D_16_
- RadScore (peritumoral) = f_Per_1 *_ c___Per_1 +_ f_Per_2 *_ c___Per_2 +_ f_Per_3 *_ c___Per_3 +_ f_Per_4 *_ c___Per_4 +_ f_Per_5 *_ c___Per_5 +_ f_Per_6 *_ c___Per_6 +_ f_Per_7 *_ c___Per_7 +_ f_Per_8 *_ c___Per_8 +_ f_Per_9 *_ c___Per_9 +_ f_Per_10 *_ c___Per_10 +_ f_Per_11 *_ c___Per_11 +_ f_Per_12 *_ c___Per_12_

1. The best features selected and their respective RadScore calculation formulas for predicting TP53 mutation.

|  | Radiomic feature | Abbreviation | Coefficient | Abbreviation |
| --- | --- | --- | --- | --- |
| 3D ROI | | | | |
|  | wavelet-LLL_firstorder_RootMeanSquared | f_3D_1_ | -0.063969998 | c_3D_1_ |
|  | wavelet-HHH_glszm_LargeAreaLowGrayLevelEmphasis | f_3D_2_ | -0.025175058 | c_3D_2_ |
|  | wavelet-HHL_glcm_MCC | f_3D_3_ | -0.06194011 | c_3D_3_ |
|  | wavelet-HHH_firstorder_Skewness | f_3D_4_ | 0.07343093 | c_3D_4_ |
|  | wavelet-LHH_glcm_Imc1 | f_3D_5_ | -0.183667174 | c_3D_5_ |
|  | original_firstorder_10Percentile | f_3D_6_ | 0.036793317 | c_3D_6_ |
|  | wavelet-HHL_gldm_LargeDependenceLowGrayLevelEmphasis | f_3D_7_ | -0.056009371 | c_3D_7_ |
|  | wavelet-HLL_firstorder_Skewness | f_3D_8_ | -0.055403109 | c_3D_8_ |
|  | wavelet-LLL_glszm_ZoneEntropy | f_3D_9_ | -0.11762495 | c_3D_9_ |
|  | wavelet-HHH_glrlm_ShortRunLowGrayLevelEmphasis | f_3D_10_ | 0.009713861 | c_3D_10_ |
|  | wavelet-HHH_glcm_ClusterShade | f_3D_11_ | 0.025020446 | c_3D_11_ |
|  | wavelet-LLH_glcm_Correlation | f_3D_12_ | -0.030698012 | c_3D_12_ |
|  | original_glcm_MaximumProbability | f_3D_13_ | 0.031735129 | c_3D_13_ |
|  | wavelet-HHH_glcm_Imc2 | f_3D_14_ | -0.216537521 | c_3D_14_ |
|  | wavelet-HLH_glszm_SizeZoneNonUniformityNormalized | f_3D_15_ | 0.041412324 | c_3D_15_ |
|  | wavelet-LLH_glrlm_LongRunHighGrayLevelEmphasis | f_3D_16_ | 0.064621018 | c_3D_16_ |
| Peritumoral RO | | | | |
|  | original_glcm_Imc1 | f_Per_1_ | 0.017925889 | c_Per_1_ |
|  | wavelet-HHL_glszm_GrayLevelNonUniformityNormalized | f_Per_2_ | 0.049488132 | c_Per_2_ |
|  | wavelet-HHH_gldm_SmallDependenceHighGrayLevelEmphasis | f_Per_3_ | 0.12837619 | c_Per_3_ |
|  | wavelet-HLL_glcm_Correlation | f_Per_4_ | 0.062036558 | c_Per_4_ |
|  | wavelet-HLH_glcm_ClusterShade | f_Per_5_ | -0.1244346 | c_Per_5_ |
|  | wavelet-LLH_glszm_SmallAreaEmphasis | f_Per_6_ | -0.054779037 | c_Per_6_ |
|  | wavelet-LHL_gldm_HighGrayLevelEmphasis | f_Per_7_ | 0.027433983 | c_Per_7_ |
|  | wavelet-LHL_glszm_LargeAreaLowGrayLevelEmphasis | f_Per_8_ | 0.07025467 | c_Per_8_ |
|  | wavelet-HHH_glrlm_RunPercentage | f_Per_9_ | -0.097128833 | c_Per_9_ |
|  | wavelet-HLH_glcm_MCC | f_Per_10_ | 0.017604458 | c_Per_10_ |
|  | wavelet-LLL_glrlm_ShortRunLowGrayLevelEmphasis | f_Per_11_ | -0.034843402 | c_Per_11_ |
|  | original_glszm_SizeZoneNonUniformityNormalized | f_Per_12_ | 0.113692071 | c_Per_12_ |

1. PIK3CA mutation

The formula for RadScores is as follows:

- RadScore (3D) = f_3D_1 *_ c___3D_1 +_ f_3D_2 *_ c___3D_2 +_ f_3D_3 *_ c___3D_3 +_ f_3D_4 *_ c___3D_4 +_ f_3D_5 *_ c___3D_5 +_ f_3D_6 *_ c___3D_6 +_ f_3D_7 *_ c___3D_7 +_ f_3D_8 *_ c___3D_8 +_ f_3D_9 *_ c___3D_9_
- RadScore (peritumoral) = f_Per_1 *_ c___Per_1 +_ f_Per_2 *_ c___Per_2 +_ f_Per_3 *_ c___Per_3 +_ f_Per_4 *_ c___Per_4 +_ f_Per_5 *_ c___Per_5 +_ f_Per_6 *_ c___Per_6 +_ f_Per_7 *_ c___Per_7 +_ f_Per_8 *_ c___Per_8 +_ f_Per_9 *_ c___Per_9 +_ f_Per_10 *_ c___Per_10 +_ f_Per_11 *_ c___Per_11 +_ f_Per_12 *_ c___Per_12_

1. The best features selected and their respective RadScore calculation formulas for predicting PIK3CA mutation.

|  | Radiomic feature | Abbreviation | Coefficient | Abbreviation |
| --- | --- | --- | --- | --- |
| 3D ROI | | | | |
|  | wavelet-HLL_ngtdm_Busyness | f_3D_1_ | 0.037784056 | c_3D_1_ |
|  | wavelet-HHL_gldm_HighGrayLevelEmphasis | f_3D_2_ | -0.106250836 | c_3D_2_ |
|  | original_shape_Flatness | f_3D_3_ | -0.115740758 | c_3D_3_ |
|  | wavelet-LHH_glcm_JointAverage | f_3D_4_ | 0.039743043 | c_3D_4_ |
|  | wavelet-HHH_glcm_JointAverage | f_3D_5_ | -0.039763569 | c_3D_5_ |
|  | wavelet-HLL_firstorder_Skewness | f_3D_6_ | -0.069799966 | c_3D_6_ |
|  | wavelet-LHH_firstorder_Skewness | f_3D_7_ | -0.138784167 | c_3D_7_ |
|  | wavelet-LHL_glszm_LowGrayLevelZoneEmphasis | f_3D_8_ | -0.141583246 | c_3D_8_ |
|  | wavelet-HHL_glszm_SmallAreaEmphasis | f_3D_9_ | -0.14216512 | c_3D_9_ |
| Peritumoral RO | | | | |
|  | wavelet-LLL_firstorder_RootMeanSquared | f_Per_1_ | -0.120968286 | c_Per_1_ |
|  | wavelet-HHL_gldm_HighGrayLevelEmphasis | f_Per_2_ | 0.104923766 | c_Per_2_ |
|  | wavelet-HLL_ngtdm_Strength | f_Per_3_ | -0.067142657 | c_Per_3_ |
|  | wavelet-HHH_glcm_Correlation | f_Per_4_ | 0.158286436 | c_Per_4_ |
|  | original_shape_Elongation | f_Per_5_ | -0.117423044 | c_Per_5_ |
|  | wavelet-LLH_glszm_LargeAreaHighGrayLevelEmphasis | f_Per_6_ | 0.002523201 | c_Per_6_ |
|  | wavelet-LLL_firstorder_Minimum | f_Per_7_ | -0.033581066 | c_Per_7_ |
|  | wavelet-LHH_glcm_Imc1 | f_Per_8_ | 0.104974639 | c_Per_8_ |
|  | wavelet-LLH_glcm_SumAverage | f_Per_9_ | 0.13194308 | c_Per_9_ |
|  | original_firstorder_Maximum | f_Per_10_ | -0.023543058 | c_Per_10_ |
|  | wavelet-LHH_glrlm_ShortRunHighGrayLevelEmphasis | f_Per_11_ | -0.199483438 | c_Per_11_ |
|  | original_glrlm_LongRunLowGrayLevelEmphasis | f_Per_12_ | -0.009467337 | c_Per_12_ |

1. ROS1 mutation

The formula for RadScores is as follows:

- RadScore (3D) = f_3D_1 *_ c___3D_1 +_ f_3D_2 *_ c___3D_2 +_ f_3D_3 *_ c___3D_3 +_ f_3D_4 *_ c___3D_4 +_ f_3D_5 *_ c___3D_5 +_ f_3D_6 *_ c___3D_6 +_ f_3D_7 *_ c___3D_7 +_ f_3D_8 *_ c___3D_8 +_ f_3D_9 *_ c___3D_9 +_ f_3D_10 *_ c___3D_10 +_ f_3D_11 *_ c___3D_11 +_ f_3D_12 *_ c___3D_12 +_ f_3D_13 *_ c___3D_13 +_ f_3D_14 *_ c___3D_14 +_ f_3D_15 *_ c___3D_15_
- RadScore (peritumoral) = f_Per_1 *_ c___Per_1 +_ f_Per_2 *_ c___Per_2 +_ f_Per_3 *_ c___Per_3 +_ f_Per_4 *_ c___Per_4 +_ f_Per_5 *_ c___Per_5 +_ f_Per_6 *_ c___Per_6 +_ f_Per_7 *_ c___Per_7 +_ f_Per_8 *_ c___Per_8 +_ f_Per_9 *_ c___Per_9 +_ f_Per_10 *_ c___Per_10 +_ f_Per_11 *_ c___Per_11 +_ f_Per_12 *_ c___Per_12 +_ f_Per_13 *_ c___Per_13 +_ f_Per_14 *_ c___Per_14 +_ f_Per_15 *_ c___Per_15 +_ f_Per_16 *_ c___Per_16 +_ f_Per_17 *_ c___Per_17 +_ f_Per_18 *_ c___Per_18 +_ f_Per_19 *_ c___Per_19 +_ f_Per_20 *_ c___Per_20 +_ f_Per_21 *_ c___Per_21_

1. The best features selected and their respective RadScore calculation formulas for predicting ROS1 mutation.

|  | Radiomic feature | Abbreviation | Coefficient | Abbreviation |
| --- | --- | --- | --- | --- |
| 3D ROI | | | | |
|  | wavelet-HHH_glcm_DifferenceAverage | f_3D_1_ | -0.286175939 | c_3D_1_ |
|  | wavelet-HHL_glcm_JointAverage | f_3D_2_ | -0.285405636 | c_3D_2_ |
|  | wavelet-LHH_glcm_Imc1 | f_3D_3_ | 0.009097784 | c_3D_3_ |
|  | wavelet-HHL_firstorder_Skewness | f_3D_4_ | -0.13069174 | c_3D_4_ |
|  | original_firstorder_Range | f_3D_5_ | 0.104516018 | c_3D_5_ |
|  | wavelet-HHH_glszm_SizeZoneNonUniformityNormalized | f_3D_6_ | 0.024050496 | c_3D_6_ |
|  | wavelet-LHL_glcm_Autocorrelation | f_3D_7_ | 0.435591305 | c_3D_7_ |
|  | wavelet-LHH_firstorder_Mean | f_3D_8_ | -0.102204022 | c_3D_8_ |
|  | wavelet-HLH_gldm_HighGrayLevelEmphasis | f_3D_9_ | 0.026101465 | c_3D_9_ |
|  | wavelet-LLH_glszm_LowGrayLevelZoneEmphasis | f_3D_10_ | -0.132518888 | c_3D_10_ |
|  | wavelet-HHH_glcm_ClusterShade | f_3D_11_ | -0.105244322 | c_3D_11_ |
|  | wavelet-LLL_firstorder_90Percentile | f_3D_12_ | 0.046977563 | c_3D_12_ |
|  | wavelet-LHL_firstorder_Skewness | f_3D_13_ | 0.054981857 | c_3D_13_ |
|  | original_gldm_GrayLevelVariance | f_3D_14_ | -0.318090931 | c_3D_14_ |
|  | wavelet-HLL_glszm_LargeAreaLowGrayLevelEmphasis | f_3D_15_ | -0.07844598 | c_3D_15_ |
| Peritumoral RO | | | | |
|  | original_shape_LeastAxisLength | f_Per_1_ | -0.039982489 | c_Per_1_ |
|  | wavelet-HLH_glcm_Autocorrelation | f_Per_2_ | 0.390216896 | c_Per_2_ |
|  | wavelet-LLH_glszm_ZoneVariance | f_Per_3_ | -0.080519248 | c_Per_3_ |
|  | wavelet-LLL_glcm_Idmn | f_Per_4_ | 0.303164856 | c_Per_4_ |
|  | wavelet-LHH_glcm_JointAverage | f_Per_5_ | 0.01608081 | c_Per_5_ |
|  | wavelet-HHH_firstorder_Skewness | f_Per_6_ | -0.041788572 | c_Per_6_ |
|  | original_firstorder_Maximum | f_Per_7_ | -0.143990032 | c_Per_7_ |
|  | wavelet-LHH_glcm_SumSquares | f_Per_8_ | 0.080162701 | c_Per_8_ |
|  | wavelet-HLL_glrlm_LongRunLowGrayLevelEmphasis | f_Per_9_ | -0.085748733 | c_Per_9_ |
|  | original_gldm_LargeDependenceLowGrayLevelEmphasis | f_Per_10_ | -0.03459923 | c_Per_10_ |
|  | wavelet-LLL_glcm_MCC | f_Per_11_ | -0.197233234 | c_Per_11_ |
|  | wavelet-LLL_ngtdm_Busyness | f_Per_12_ | -0.142789615 | c_Per_12_ |
|  | wavelet-HLH_glcm_MCC | f_Per_13_ | -0.01579889 | c_Per_13_ |
|  | wavelet-HHL_glcm_Correlation | f_Per_14_ | 0.168269745 | c_Per_14_ |
|  | wavelet-HHL_glszm_LargeAreaLowGrayLevelEmphasis | f_Per_15_ | -0.009603748 | c_Per_15_ |
|  | wavelet-HHH_glrlm_ShortRunHighGrayLevelEmphasis | f_Per_16_ | -0.140939134 | c_Per_16_ |
|  | wavelet-LLH_glszm_SmallAreaLowGrayLevelEmphasis | f_Per_17_ | 0.123485216 | c_Per_17_ |
|  | wavelet-HHH_glszm_SmallAreaEmphasis | f_Per_18_ | 0.014583411 | c_Per_18_ |
|  | original_shape_SurfaceVolumeRatio | f_Per_19_ | 0.110831603 | c_Per_19_ |
|  | wavelet-HHH_firstorder_Mean | f_Per_20_ | -0.185555182 | c_Per_20_ |
|  | wavelet-LLH_ngtdm_Strength | f_Per_21_ | -0.260765487 | c_Per_21_ |

1. PD-1/PD-L1 expression

The formula for RadScores is as follows:

- RadScore (3D) = f_3D_1 *_ c___3D_1 +_ f_3D_2 *_ c___3D_2 +_ f_3D_3 *_ c___3D_3 +_ f_3D_4 *_ c___3D_4 +_ f_3D_5 *_ c___3D_5 +_ f_3D_6 *_ c___3D_6 +_ f_3D_7 *_ c___3D_7 +_ f_3D_8 *_ c___3D_8 +_ f_3D_9 *_ c___3D_9 +_ f_3D_10 *_ c___3D_10 +_ f_3D_11 *_ c___3D_11 +_ f_3D_12 *_ c___3D_12_
- RadScore (peritumoral) = f_Per_1 *_ c___Per_1 +_ f_Per_2 *_ c___Per_2 +_ f_Per_3 *_ c___Per_3 +_ f_Per_4 *_ c___Per_4 +_ f_Per_5 *_ c___Per_5 +_ f_Per_6 *_ c___Per_6 +_ f_Per_7 *_ c___Per_7_

1. The best features selected and their respective RadScore calculation formulas for predicting PD-1/PD-L1 expression.

|  | Radiomic feature | Abbreviation | Coefficient | Abbreviation |
| --- | --- | --- | --- | --- |
| 3D ROI | | | | |
|  | wavelet-HHH_glrlm_HighGrayLevelRunEmphasis | f_3D_1_ | -0.007087995 | c_3D_1_ |
|  | wavelet-HHH_glszm_SizeZoneNonUniformity | f_3D_2_ | -0.002773063 | c_3D_2_ |
|  | wavelet-HLH_firstorder_Skewness | f_3D_3_ | -0.072508531 | c_3D_3_ |
|  | wavelet-HHH_firstorder_Mean | f_3D_4_ | -0.18921138 | c_3D_4_ |
|  | wavelet-HLH_firstorder_Mean | f_3D_5_ | -0.010628738 | c_3D_5_ |
|  | original_shape_Flatness | f_3D_6_ | 0.087369714 | c_3D_6_ |
|  | wavelet-LLL_gldm_HighGrayLevelEmphasis | f_3D_7_ | -0.016437671 | c_3D_7_ |
|  | original_glcm_Correlation | f_3D_8_ | -0.037102337 | c_3D_8_ |
|  | wavelet-LLH_gldm_DependenceEntropy | f_3D_9_ | -0.098463811 | c_3D_9_ |
|  | wavelet-LLH_glcm_ClusterShade | f_3D_10_ | 0.025786552 | c_3D_10_ |
|  | wavelet-LLL_firstorder_90Percentile | f_3D_11_ | -0.067367604 | c_3D_11_ |
|  | wavelet-LHL_glcm_ClusterShade | f_3D_12_ | -0.080292126 | c_3D_12_ |
| Peritumoral RO | | | | |
|  | original_shape_Flatness | f_Per_1_ | 0.081943389 | c_Per_1_ |
|  | wavelet-HLH_gldm_SmallDependenceLowGrayLevelEmphasis | f_Per_2_ | -0.014840131 | c_Per_2_ |
|  | wavelet-LHH_glcm_Imc1 | f_Per_3_ | 0.050637097 | c_Per_3_ |
|  | wavelet-HLL_firstorder_Mean | f_Per_4_ | 0.10854203 | c_Per_4_ |
|  | wavelet-HLL_firstorder_Range | f_Per_5_ | 0.012068842 | c_Per_5_ |
|  | wavelet-HHL_firstorder_Skewness | f_Per_6_ | 0.061954736 | c_Per_6_ |
|  | wavelet-LHL_firstorder_Kurtosis | f_Per_7_ | -0.033428505 | c_Per_7_ |

1. The prediction of prognostic risk factors, gene mutations, and immunoexpression outcomes in the training cohort.

|  | Model | Classifier | AUC (95% CI) | Accuracy (%) | Precision (%) | Recall (%) | F1 score (%) |
| --- | --- | --- | --- | --- | --- | --- | --- |
| Prognostic risk factors | | | | | | | |
| LVI | Radiomics-Score | NB | 0.866 [0.855 - 0.875] | 80.4 | 82.4 | 80.4 | 79.8 |
|  | Deep-CT | MLP | 0.833 [0.821 - 0.844] | 79.2 | 79.0 | 80.5 | 76.4 |
|  | Deep-RadScore | LDA | 0.919 [0.911 - 0.928] | 84.6 | 85.4 | 84.6 | 84.3 |
| PI | Radiomics-Score | SVM | 0.906 [0.900 - 0.912] | 83.5 | 83.6 | 83.6 | 83.5 |
|  | Deep-CT | RF | 0.893 [0.887 - 0.900] | 78.1 | 78.2 | 78.2 | 78.1 |
|  | Deep-RadScore | SVM | 0.942 [0.937 - 0.946] | 86.9 | 87.0 | 87.0 | 86.9 |
| T staging | Radiomics-Score | NB | 0.839 [0.829 - 0.849] | 75.9 | 76.1 | 75.9 | 75.8 |
|  | Deep-CT | LDA | 0.863 [0.857 - 0.870] | 78.7 | 78.8 | 78.8 | 78.7 |
|  | Deep-RadScore | RF | 0.942 [0.939 - 0.946] | 85.0 | 85.4 | 85.1 | 84.9 |
| Gene mutations | | | | | | | |
| EGFR | Radiomics-Score | SVM | 0.851 [0.840 - 0.863] | 78.9 | 79.6 | 79.0 | 78.4 |
|  | Deep-CT | NB | 0.848 [0.836 - 0.860] | 79.3 | 80.6 | 80.5 | 79.2 |
|  | Deep-RadScore | RF | 0.892 [0.762 - 0.782] | 84.1 | 84.5 | 84.2 | 84.0 |
| KRAS | Radiomics-Score | MLP | 0.863 [0.854 - 0.873] | 78.3 | 79.5 | 78.8 | 77.9 |
|  | Deep-CT | LR | 0.845 [0.685 - 0.704] | 77.2 | 77.5 | 77.2 | 77.1 |
|  | Deep-RadScore | LDA | 0.914 [0.766 -0.782] | 83.1 | 83.2 | 83.1 | 81.0 |
| ALK | Radiomics-Score | RF | 0.844 [0.835 - 0.854] | 78.4 | 80.3 | 78.5 | 78.1 |
|  | Deep-CT | RF | 0.813 [0.804 - 0.823] | 75.4 | 77.5 | 77.8 | 74.8 |
|  | Deep-RadScore | RF | 0.920 [0.913 - 0.937] | 83.7 | 81.2 | 78.9 | 81.3 |
| TP53 | Radiomics-Score | KNN | 0.844 [0.834 - 0.853] | 76.5 | 76.9 | 76.6 | 76.3 |
|  | Deep-CT | SVM | 0.863 [0.855 - 0.871] | 79.4 | 79.6 | 79.6 | 79.4 |
|  | Deep-RadScore | LDA | 0.920 [0.913 - 0.926] | 84.3 | 84.5 | 84.4 | 84.3 |
| PIK3CA | Radiomics-Score | KNN | 0.877 [0.869 - 0.885] | 81.7 | 78.9 | 78.7 | 78.6 |
|  | Deep-CT | NB | 0.845 [0.835 - 0.855] | 78.0 | 76.1 | 76.1 | 76.9 |
|  | Deep-RadScore | LDA | 0.933 [0.926 - 0.939] | 85.3 | 85.9 | 85.4 | 85.2 |
| ROS1 | Radiomics-Score | MLP | 0.833 [0.823 - 0.842] | 76.4 | 76.5 | 76.5 | 76.3 |
|  | Deep-CT | RF | 0.858 [0.849 - 0.867] | 78.1 | 79.9 | 78.5 | 77.6 |
|  | Deep-RadScore | NB | 0.928 [0.921 - 0.935] | 85.5 | 86.7 | 85.6 | 85.3 |
| Immunoexpression | | | | | | | |
| PD-1/PD-L1 | Radiomics-Score | NB | 0.861 [0.845 - 0.877] | 80.6 | 80.3 | 80.9 | 80.4 |
|  | Deep-CT | LDA | 0.851 [0.834 - 0.868] | 79.2 | 80.5 | 80.4 | 79.1 |
|  | Deep-RadScore | LR | 0.919 [0.904 - 0.933] | 86.8 | 87.0 | 86.9 | 86.6 |
| Abbreviations: CI, confidence intervals; SVM, support vector machine; KNN, k-nearest neighbors; RF, random forests; NB, naive Bayes classifier; LR, logistic regression; MLP, multilayer perceptron; LDA, linear discriminant analysis. | | | | | | | |

1. Introspection of Deep-RadScore

Fig. S3 to Fig. S5 illustrates the influence of individual feature values on prognostic risk factors, gene mutations, and PD-1/PD-L1 expression levels, respectively.

1. Visualization of Deep-CT network

Fig. S6 illustrates an example of the specific learning focus of the Deep-CT network on a particular classification target.

1. Patient recruitment workflow. A total of 508 out of 908 patients were included in the study based on the screening criteria. The included patients underwent chest CT and provided complete clinicopathologic information required for the study.

1. The detailed structure of the network. *Note: the yellow shading (Fc1) refers to the deep network feature output layer.

1. Comparison of how feature values impact the results of Deep-RadScore in predicting LVI, PI, and T4 staging. LVI, Abbreviations: lymphovascular invasion; PI, pleural invasion.

1. Comparison of how feature values impact the results of Deep-RadScore in predicting EGFR mutation, KRAS mutation, ALK mutation, TP53 mutation, PIK3CA mutation, and ROS1 mutation.

1. Comparison of how feature values impact the results of Deep-RadScore in predicting PD-1/PD-L1 expression.

1. Visualizing a patient example, where red areas correspond to larger weights, decoded using the right-side color bar.

References

1. Zhang GJ, Cao YT, Zhang J, Ren JL, Zhao ZY, Zhang XD, et al. Predicting EGFR mutation status in lung adenocarcinoma: development and validation of a computed tomography-based radiomics signature. Am J Cancer Res. 2021;11:546-+.
2. Kawazoe Y, Shiinoki T, Fujimoto K, Yuasa Y, Hirano T, Matsunaga K, et al. Comparison of the radiomics-based predictive models using machine learning and nomogram for epidermal growth factor receptor mutation status and subtypes in lung adenocarcinoma. Phys Eng Sci Med. 2023.
3. Yang XY, Liu M, Ren YH, Chen H, Yu PX, Wang SY, et al. Using contrast-enhanced CT and non-contrast-enhanced CT to predict EGFR mutation status in NSCLC patients-a radiomics nomogram analysis. Eur Radiol. 2022;32:2693-703.
4. Shiri I, Amini M, Nazari M, Hajianfar G, Avval AH, Abdollahi H, et al. Impact of feature harmonization on radiogenomics analysis: Prediction of EGFR and KRAS mutations from non-small cell lung cancer PET/CT images. Comput Biol Med. 2022;142.
5. Xie XJ, Liu SY, Chen JY, Zhao Y, Jiang J, Wu L, et al. Development of unenhanced CT-based imaging signature for BAP1 mutation status prediction in malignant pleural mesothelioma: Consideration of 2D and 3D segmentation. Lung Cancer. 2021;157:30-9.
6. Arefan D, Chai RM, Sun M, Zuley ML, Wu SD. Machine learning prediction of axillary lymph node metastasis in breast cancer: 2D versus 3D radiomic features. Med Phys. 2020;47:6334-42.
7. Liao CY, Lee CC, Yang HC, Chen CJ, Chung WY, Wu HM, et al. Predicting survival after radiosurgery in patients with lung cancer brain metastases using deep learning of radiomics and EGFR status. Phys Eng Sci Med. 2023.
8. Ortiz-Ramon R, Larroza A, Arana E, Moratal D. A Radiomics Evaluation of 2D and 3D MRI Texture Features to Classify Brain Metastases from Lung Cancer and Melanoma. P Ann Int Ieee Embs. 2017:493-6.
9. Avard E, Shiri I, Hajianfar G, Abdollahi H, Kalantari KR, Houshmand G, et al. Non-contrast Cine Cardiac Magnetic Resonance image radiomics features and machine learning algorithms for myocardial infarction detection. Comput Biol Med. 2022;141.
10. Alhussaini AJ, Steele JD, Nabi G. Comparative Analysis for the Distinction of Chromophobe Renal Cell Carcinoma from Renal Oncocytoma in Computed Tomography Imaging Using Machine Learning Radiomics Analysis. Cancers. 2022;14.
11. Hu YH, Xie CY, Yang H, Ho JWK, Wen J, Han LJ, et al. Assessment of Intratumoral and Peritumoral Computed Tomography Radiomics for Predicting Pathological Complete Response to Neoadjuvant Chemoradiation in Patients With Esophageal Squamous Cell Carcinoma. Jama Netw Open. 2020;3.
